# Practical and technical aspects for the 3D scanning of lithic artefacts using micro-computed tomography techniques and laser light scanners for subsequent geometric morphometric analysis. Introducing the StyroStone protocol

**DOI:** 10.1371/journal.pone.0267163

**Published:** 2022-04-21

**Authors:** Dominik Göldner, Fotios Alexandros Karakostis, Armando Falcucci

**Affiliations:** 1 Department of Palaeoanthropology, Institute of Archaeological Science, University of Tübingen, Tübingen, Germany; 2 DFG (Deutsche Forschungsgemeinschaft) Center for Advanced Studies “Words, Bones, Genes, Tools,” Eberhard Karls University of Tübingen, Tübingen, Germany; 3 Department of Early Prehistory and Quaternary Ecology, Schloss Hohentübingen, University of Tübingen, Tübingen, Germany; New York University, UNITED STATES

## Abstract

Here, we present a new method to scan a large number of lithic artefacts using three-dimensional scanning technology. Despite the rising use of high-resolution 3D surface scanners in archaeological sciences, no virtual studies have focused on the 3D digitization and analysis of small lithic implements such as bladelets, microblades, and microflakes. This is mostly due to difficulties in creating reliable 3D meshes of these artefacts resulting from several inherent features (i.e., size, translucency, and acute edge angles), which compromise the efficiency of structured light or laser scanners and photogrammetry. Our new protocol *StyroStone* addresses this problem by proposing a step-by-step procedure relying on the use of micro-computed tomographic technology, which is able to capture the 3D shape of small lithic implements in high detail. We tested a system that enables us to scan hundreds of artefacts together at once within a single scanning session lasting a few hours. As also bigger lithic artefacts (i.e., blades) are present in our sample, this protocol is complemented by a short guide on how to effectively scan such artefacts using a structured light scanner (Artec Space Spider). Furthermore, we estimate the accuracy of our scanning protocol using principal component analysis of 3D Procrustes shape coordinates on a sample of meshes of bladelets obtained with both micro-computed tomography and another scanning device (i.e., Artec Micro). A comprehensive review on the use of 3D geometric morphometrics in lithic analysis and other computer-based approaches is provided in the introductory chapter to show the advantages of improving 3D scanning protocols and increasing the digitization of our prehistoric human heritage.

## Introduction

Traditional typological and metric analyses of lithic artefacts are commonly used in archaeology to study both intra- and inter-site assemblage variability as well as spatiotemporal development in relation to human evolution and an individual’s ability to craft and use stone tools [1–4]. While typology-based approaches are affected by the analyst’s experience, low reproducibility, and classification biases [2], traditional metric analysis is based on more objective features: two-dimensional linear distance measurements, ratios, and angles. These measurements can be reliably and easily collected in a quantitative manner; however, they are still constrained by some inherent methodological boundaries [2, 5]. One of the major drawbacks is the relatively low number of well-defined measurements used to quantify the dimensions of artefacts. Additionally, measurements cannot be used to establish any spatial relationship between measured distances. As a consequence, analysis mostly focuses on size characteristics, whereas information about the shape and form of an object are limited and mostly assessed using qualitative approaches [5–9].

Geometric morphometrics (GM) is an effective alternative to quantitatively capture and preserve shape and form information throughout statistical analysis [10, 11]. GM analysis has been increasingly used in the natural sciences since the second half of the last century, proving to be a valuable set of statistical tools to better understand morphological variability and evolution within and between species [11, 12]. Over the last decades, a vast number of studies have empirically demonstrated the advantages of GM analysis over traditional linear morphometrics beyond biological research, including the study of material culture in archaeology [e.g., 13, 14–16]. Today, GM analysis of lithic assemblages has already outgrown its novelty status and many recent research projects make use of this approach to study shape- and form-related techno-functional aspects of tool use and edge resharpening, technological variability, as well as differences between artefact types [see among others: 1, 9, 17, 18–37].

The most common data format in GM are landmarks and semilandmarks. These measurements consist of a set of either two- (2D) or three-dimensional (3D) Cartesian coordinate data, which accurately capture the relational shape or form information of a specimen [5]. Useful open-source software solutions to manually collect (semi)landmarks are, among others, the 3D mesh editor program MeshLab [38], Morphodig [39], the Tps software series [40] and the R [41] package *geomorph* [42]. Traditional landmark data points as defined by Bookstein [43], depend on precisely defined and clearly identifiable homologous points that have to be present on all specimens across the entire sample. These traditional so-called Bookstein landmarks, however, often correspond to the endpoints of linear measurements in traditional 2D morphometrics as mentioned above and, therefore, display the same limitations [2]. Furthermore, as stone tools are human-made objects and not organically grown, it is in theory and practice impossible to establish geometric correspondence [43], which is typically required in most traditional applications of GM [2]. The concept of semilandmarks can be used to effectively overcome this obstacle [2, 7, 44].

Semilandmarks are not constrained by clear-cut definitions regarding their locations and can, therefore, be placed practically anywhere. This allows the analysts to capture homologous surfaces and/or curvature outlines, which are not or are rarely represented by traditional landmark configurations. As opposed to Bookstein landmarks, it is recommended to ‘slide’ semilandmarks after their digitization [44]. Although this is not always necessary, the sliding process helps to remove the influence of the arbitrary spacing of manually digitized points and to establish geometric correspondence between semilandmark configurations of homologous structures across specimens. In subsequent GM analysis, semilandmark data points are statistically analysed like fixed landmarks [7, 44]. Semilandmarks can either be placed manually using one of the aforementioned programs or with (semi)automatic software such as the open-source package *Artifact Geomorph Toolbox 3D* (AGMT3-D) which has been specially developed for the analysis of lithic artefacts [20].

AGMT3-D first aligns the position and orientation of the lithic scans and then automatically places surface and curve semilandmarks over the entire surface and outline of 3D artefact meshes. The user is only required to check and adjust the positioning of each artefact in the 3D space to allow for the digitization of geometrically correspondent semilandmarks across the whole sample and to specify a semilandmark configuration. The software will then automatically place and save the collected landmark data for further statistical analysis.

Once the (semi)landmark data has been collected it can be subjected to multivariate GM analyses including Generalized Procrustes Analysis, Principal Component Analysis, Partial Least Square Analysis, Linear Discriminant Function, and Multi Regression Analysis, among others [5]. Different programs exist to conduct these kinds of analyses, such as the statistical programming software environment R [41], for which special GM packages like *geomorph* [42] and *morpho* [45] were developed. There are also programs that come with inbuilt GM functions and more intuitive graphic user interfaces, such as PAST [46]. Likewise, AGMT3-D has a wide range of standard analytical GM functions [20]. We encourage the reader to see Mitteroecker and Gunz [5] and Brande and Saragusti [47] for additional information on GM and Shott and Trail [8] and Okumura and Araujo [10] for an in-depth discussion on GM as applied to stone tools.

Despite the increasing application of GM analyses to assess the variability of lithic artefacts, more effort is required to reach higher scientific standards for scanning and recording 3D meshes, as well as subsequent data handling, standardisation of landmark configurations for comparability and reproducibility, and the sharing of lab protocols and raw data to support Open Science compliant practices [48]. As data acquisition, handling, and analytical procedures are much more complex with 3D GM approaches compared to traditional linear morphometrics, there is increasing demand for the sharing of methods and data, which ensures transparency for independent research validation and cross-study comparability. In the following paragraphs, we will focus solely on the 3D scanning process for lithic artefacts, as this usually represents the preliminary step for any 3D GM analysis. We will not discuss lithic photography [e.g., 37] or drawing [e.g., 35], which could alternatively be used for the collection of 2D semilandmarks of a lithic’s outline [5]. The increasing accessibility to optical scanners and other devices, as well as increasing interest by archaeologists in new computerized methods [49, 50], has made such devices more accessible to archaeological institutes worldwide.

There are numerous factors contributing to the final quality of 3D models which are particularly apparent when comparing the different scanning techniques, 3D registration, and reconstruction methods available [51]. One of the main problems with 3D data acquisition of lithic artefacts is that they are usually found in large quantities during archaeological excavations. In many cases, it is considered time consuming and impractical to scan lithic artefacts individually when designing a 3D GM analysis. Furthermore, many Palaeolithic assemblages are dominated by very small lithic implements that sometimes have a maximum linear dimension of 10 mm (e.g., bladelets, microblade, and small flakes). The considerably small dimensions of these artefacts can hinder the use of common surface scanning procedures (i.e., structured light and laser scanning) or image-based, close-range photogrammetry. In these circumstances, accurate detection of the often very sharp edges of a lithic might fail, resulting in a negative influence on the subsequent shape analysis. Likewise, semi-transparent, translucent, as well as reflective, shiny surfaces can also lead to scanning problems depending on the material properties of the scanned artefact. Special aerosol sprays that are easy to remove and usually contain talc particles, alcohol, and acetone have been developed to temporally dull the surface to help optical scanners detect and match geometry features more efficiently. However, it is not always feasible to expose archaeological materials to chemicals, as it might affect use-wear and residue analyses. If lithics are not intended for further analyses, it is suggested, nevertheless, to test beforehand whether the spray has any effect on labelling and other surface features [21, 51].

To close some of the aforementioned gaps in 3D data acquisition of especially large lithic assemblages, we provide a comprehensive, step-by-step protocol relying on the use of micro-computed tomographic (Micro-CT) scanners and structured light scanners that will allow archaeologists to increase the utility of computerized methods in the study of stone tools with variable morpho-metric attributes.

## Materials and methods

The different challenges in 3D scanning discussed in this paper relate mostly to inter-dependent factors such as the large number of artefacts that need to be scanned, their size, the acute edge-angle, and their translucency, which can cause several problems when using photogrammetry and surface scanners. However, these issues can be avoided when using a Micro-CT scanner. The *StyroStone* protocol described in this peer-reviewed article is published on protocols.io, doi.org/10.17504/protocols.io.4r3l24d9qg1y/v2 [52], and is included for printing as supporting information file 1 with this article. The protocol shows step-by-step how to rapidly scan large numbers of small lithic artefacts and extract 3D meshes using a Micro-CT scanner (i.e., a Phoenix v-tome-x s model by General Electronics MCC, Boston MA) and various software programs for postprocessing, such as Avizo Lite (Thermo Fischer Scientific Inc., Berlin; version 9.2.0) and Artec Studio Professional (Artec Inc., Luxembourg; version 15.0.3.425). Furthermore, the protocol shows how to effectively scan larger lithic artefacts (above ~35 mm in maximum length) using the structured blue light Artec Space Spider Scanner (https://www.artec3d.com/). This protocol has been successfully applied to scan several hundred blades and bladelets from the Protoaurignacian layers at Fumane Cave in north-eastern Italy [53]. All 3D models in both .ply and .wrl formats are archived on Zenodo and are free to be used with proper attribution [54]. The Protoaurignacian lithic assemblage from Fumane Cave [55] is permanently stored at the University of Ferrara, Dipartimento di Studi Umanistici, Sezione di Scienze Preistoriche e Antropologiche, Corso Ercole I d’Este, 32, I-44100 Ferrara, Italy. Lithics had not individual numbers, but were labeled according to the site of provenience (RF, that stands for Riparo di Fumane), square, sub-square, and archaeological layer. No permits were required for the described study, which complied with all relevant regulations.

**Fig 1:**
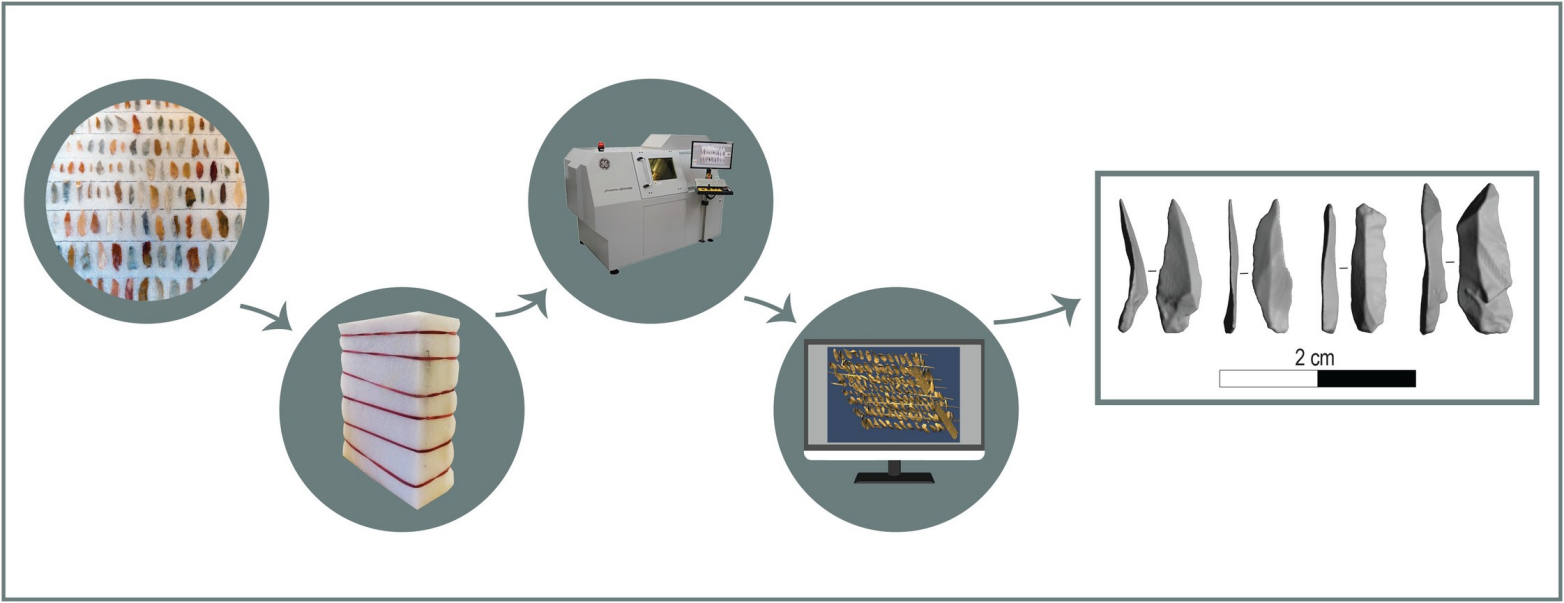
General working pipeline of the *StyroStone* protocol. The first two phases (starting from the left) summarize the insertion of the lithic artifact in the Styrofoam body, followed by the Micro-CT scanning, the 3D reconstruction and postprocessing of the scans. Finally, the last frame displays a sample of extracted 3D models of bladelets.

Fig 1 schematically shows the general structure of the protocol working pipeline. Using a Micro-CT scanner and our protocol, we were able to scan up to 220 bladelets at once in a relatively short period of time (ca. 2 hours). To do that, we prepared a Styrofoam body where artifacts were arranged in multiple rows in both faces of the Styrofoam body (note that the protocol also describes the steps necessary to prepare such Styrofoam bodies). Although the scanning procedure could be accomplished over a short period of time, the subsequent extraction and separation of the individual artefacts in Avizo and Artec Studio Professional was time consuming. However, in comparison to other methods of scanning small artifacts, this extra time is negligible. Our protocol is particularly suitable to this contingency as it minimizes the number of scans while maximizing the number of artefacts scanned in a single session. In this study, the Phoenix Micro-CT scanner was operated by a lab technician and the resolution used was 140 microns. Resolution largely depends on the distance between the source and the target. Even though the Phoenix Micro-CT scanner is technically capable of producing models with greater resolution, our tests suggest that the used resolution represents a good balance for scanning a large number of artefacts at once, while avoiding distinguishable inaccuracies in the resulting 3D surface shapes. Our protocol can be applied when using other Micro-CT scanner models although the scanning procedure and commands might be different. The final CT scans had around 1,000 image slices and a total file size of approximately 25 to 50 gigabytes. After 3D reconstruction and extraction of individual artefacts, each model was about 200 to 2,000 kilobytes large depending on its physical size. The subsequent extraction and 3D reconstruction steps will overall remain identical if the same software is applied (i.e., Avizo, Artec Studio, and MeshLab). Likewise, all described 3D model reconstruction, extraction, and postprocessing steps can also be achieved by other software such as 3D-Slicer (https://www.slicer.org/) and Morphodig [39], although some minor adjustments may be necessary.

While it is true that most archaeological institutions do not own a Micro-CT scanner, mainly due to the purchase prices and their costly maintenance, many well-resourced natural science faculties do own Micro-CT scanners and often grant access upon request [56]. Medical CT devices, which are far more common and can be found in most larger clinics as well as local radiology offices, cannot be considered a proper solution for small lithic artefacts due to the rather low resolution, typically between 0.5 to 0.3 mm, in comparison with Micro-CT scanners. Besides their overall high costs, most Micro-CT scanners are stationary and cannot be transported elsewhere. This limits the application of Micro-CT scanning in areas without reliable and/or affordable access to scanning devices. In this case, it may be useful to discuss the transfer of archaeological materials within and between countries, with respect to the potential logistical, legal, and political challenges [51].

As mentioned above, we used an Artec Space Spider scanner for larger objects, mainly to minimize the number of Micro-CT scans needed. Based on our experience, we suggest using this scanner for lithic artefacts that are greater than ~35 mm in the maximum linear dimension and ~2.5 mm in thickness. While it might still be possible to scan slightly smaller artefacts with an Artec Spider scanner, size, opacity, and edge sharpness may increase the likelihood of scanning errors.

The Artec Space Spider Scanner has an accuracy of up to 0.05 mm and an ultra-high resolution of up to 0.1 mm, which makes it an ideal scanner for small to medium-large objects (https://www.artec3d.com/portable-3d-scanners/artec-spider). Another scanner of the same company specially designed for small to very small objects is the Artec Micro with an accuracy of up to 0.01 mm and a resolution of up to 0.029 mm (https://www.artec3d.com/portable-3d-scanners/artec-micro). The efficiency of the Artec Micro in scanning small lithic artifacts has been tested by one of us (AF) and the related step-by-step protocol is available on protocols.io [57].

Pricewise, the Artec scanners, which come together with the software Artec Studio Professional, can be considered a more cost-effective solution compared to Micro-CT scanners, considering its wide applicability, high resolution, and overall user-friendliness. The Space Spider model can be transported directly to sites for fieldwork activities using an external battery. It should however be kept in mind that its use in warmer and more humid climates and the exposure to dust particles, which are common problems on archaeological sites, might cause technical damage to the scanner. Next to the scanner itself, a powerful laptop, a number of cables, calibration tools, a protective transport case, and optionally a turntable are also required. The latter can generally be recommended in 3D scanning processes as it accelerates and comforts the procedure. Small, non-automatic turntables can be purchased for a very low price. The artefact only needs to be secured on the turntable using, for instance, a modelling clay that does not leave traces when removed. As already suggested by [21], an effective way to enhance tracking during the scanning process is to use a white turntable (this can also be achieved by covering the turntable with white cardboard) with several drawn reference circles in red and blue. Note that a wide range of other light- and laser-based surface scanners exist on the market, which might be equally suitable for scanning archaeological materials; however, none of these have been tested or applied by us.

## Validation study

**Fig 2:**
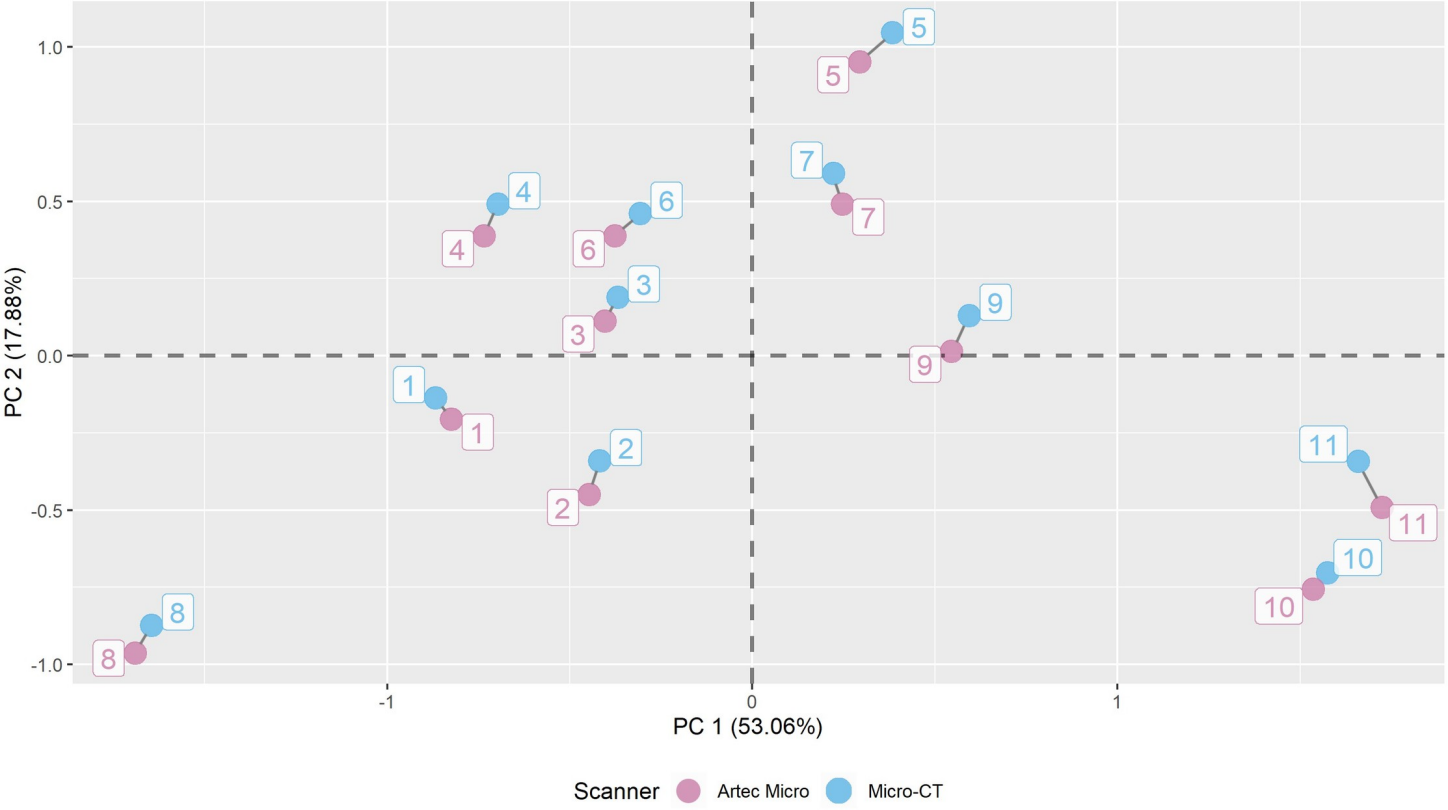
Error shape-PCA of an experimental sample of bladelets (n = 11) obtained from Micro-CT (blue) and Artec Micro (pink) scanners. Together, PC1 and PC2 explain 70.94% of the captured 3D shape variation. The R script used to produce this plot is available in the research compendium on Zenodo [67].

Although only occasionally done for 3D meshes of lithic artefacts [58], an increasing number of validation studies tested and compared the accuracy of different scanners and 3D registration methods, also including Micro-CT and structured-light surface scanners [51, 56, 59–62]. So far, both scanning technologies have been shown to provide highly accurate 3D models [63, 64]. To demonstrate the accuracy achieved by our protocol, we conducted a GM shape analysis of a small sample (n = 11) of randomly selected and experimentally produced bladelets. To do so, we scanned all artifacts twice, using both a Micro-CT scanner, following the *StyroStone* protocol, and an Artec Micro scanner following the *MicroStone* protocol [57]. After scanning, all 3D models in .wrl format were loaded into AGMT3-D v3.1 [20] to standardize their position and orientation and to automatically place a total of 400 semilandmarks using a 20x10 grid on both artefacts’ surfaces. The semilandmark coordinates were then subjected to Generalized Procrustes Analysis (GPA) to produce Procrustes shape coordinate data that are invariant of size, position and rotation [5]. An error shape-PCA was then performed on the Procrustes variables to obtain PC scores. All PC scores were then exported as .csv file and loaded into R (version 4.0.3) to create a PCA plot using the packages *ggplot2* v3.3.5 [65] and *ggrepel* v0.9.1 [66]. 3D models, as well as raw semilandmark coordinates, generated datasets, and R scripts are available in the associated research compendium available on Zenodo [67]. Fig 2 shows the shape distribution of all scanned specimens in a bivariate plot of the first two principal components (PCs), that cumulatively explain 70.94% of the captured shape variance. Here, each pair of bladelets from both scanning methods clusters closely together. This indicates that the variance in shape between repetitions is much smaller than the overall variance observed for both scanning methods. In order to further test the differences across the two groups, we performed a nonparametric MANOVA (i.e., PERMANOVA) on the first five PCs (explaining the 90% of total variance) using 10,000 repetitions in PAST v4.03 [46]. We find no significant variation between the blanks scanned with the Micro-CT and the Artec Micro (F = 0.04, *p* = 1). This finding allows us to conclude that both Micro-CT and Artec Micro produce overall similar 3D models that can be effectively used to collect semilandmarks for 3D GM analysis. Furthermore, this estimation of disparity between two different scanning methods shows the merits of exploring and comparing different scanning devices and artefacts’ inherent attributes. Future research will thus provide additional information to estimate these interrelated variables to enhance scientific standards of accuracy and reproducibility.

## Expected results

The last few decades of Palaeolithic research have seen an exponential interest in computer-based methods applied to archaeological research [49], which has resulted in several studies that have explored material culture variability and human behaviour with a higher degree of objectivity. In this fervent and stimulating research framework, the accessibility to new technologies that allow for the fast reconstruction of 3D meshes of archaeological artefacts has resulted in the application of new and powerful means of analysis. Besides the numerous papers that have explored the use of GM analysis to stone artefacts discussed in this paper, the use of 3D technology has also allowed researchers to quantify reduction intensity on cores [e.g., 68], accurately measure angles between surfaces of bone and stone tools [e.g., 69, 70], and assess knapping skills with the use of virtual refittings [e.g., 71, 72], among other applications.

We hope that our new method to scan large quantities of lithic artefacts with Micro-CT scanners and the following generation of 3D meshes will enable researchers to conduct more detailed studies on small-sized stone tool technologies such as bladelets and microliths that are still challenging to accurately scan with other scanners. This will permit researchers to focus not only on macro-tools but also to explore the small-sized component that characterizes many late Pleistocene and early Holocene technocomplexes. We believe that 3D scanning and 3D GM will contribute to a better understanding of our prehistory, although these methods should always be considered complementary tools to more traditional methods of analysis [21, 73]. Lastly, 3D scanning will allow researchers to create open-access repositories of archaeological artefacts that can be accessed worldwide, encouraging more collaborative studies across academic institutions and enhancing Open Science practices in archaeological sciences.

## Supporting information

S1 File Step-by-step protocol entitled ‘StyroStone: A protocol for scanning and extracting three-dimensional meshes of stone artefacts using Micro-CT scanners’. Also available on protocols.io (doi.org/10.17504/protocols.io.4r3l24d9qg1y/v2).
(PDF)
