# Supplementary material for: Practical and technical aspects for the 3D scanning of lithic artefacts using micro-computed tomography techniques and laser light scanners for subsequent geometric morphometric analysis. Introducing the StyroStone protocol
Source: PLoS One. 2022 Apr 21;17(4):e0267163. doi: 10.1371/journal.pone.0267163 (PMC9022823; doi:10.1371/journal.pone.0267163)
Supplement: S1 File — Also available on protocols.io (doi.org/10.17504/protocols.io.4r3l24d9qg1y/v2). (PDF) [file pone.0267163.s001.pdf]

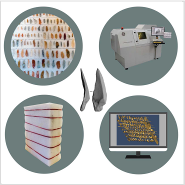

2
Mar 18, 2022

StyroStone: A protocol for scanning and extracting three-dimensional meshes of stone artefacts using Micro-CT scanners V.2

Dominik Göldner[1], Fotios Alexandros Karakostis[2], Armando Falcucci[3]

[1]Department of Paleoanthropology, University of Tübingen, Institute of Archaeological Science, D-72074 Tübingen, Germany;
[2]DFG (Deutsche Forschungsgemeinschaft) Center for Advanced Studies "Words, Bones, Genes, Tools," Eberhard Karls University of Tübingen, Tübingen, Germany;
[3]Department of Early Prehistory and Quaternary Ecology, University of Tubingen, Schloss Hohentubingen, D-72070 Tubingen, Germany

1

dx.doi.org/10.17504/protocols.io.4r3l24d9qg1y/v2

3D Lithics StyroStone

Dominik Göldner

This protocol presents the first detailed step-by-step pipeline for the 3D scanning and post processing of large batches of lithic artefacts using a micro-computed tomography (micro-CT) scanner (i.e., a Phoenix v-tome-x S model by General Electronics MCC, Boston MA) and an Artec Space Spider scanner (Artec Inc., Luxembourg). This protocol was used to scan more than 700 lithic artefacts from the Protoaurignacian layers at Fumane Cave in north-eastern Italy (https://doi.org/10.5281/zenodo.6362150).

For this study several costly scanners and proprietary software packages were employed. Although it is not easy to find a low-budget alternative for the scanners, it is possible to use free and open-source software programs, such as 3D-Slicer (https://www.slicer.org/) or MorphoDig (https://morphomuseum.com/morphodig), to process CT data as well as MeshLab (Cignoni et al. 2008) to interact with the 3D models in general. However, if alternative software is used, the steps and their order described in this protocol might diverge significantly.

A cost-effective alternative to create 3D models is digital photogrammetry using commercial cameras and freely available software like Meshroom (https://alicevision.org). Although photogrammetry is an affordable technique to create accurate 3D models of objects, this method might not be useful when scanning large batches of artefacts, as it will require a lot of computation time and processing capacity. Likewise, it could be difficult to generate accurate 3D models of very small and/or detailed tool shapes using 3D surface scanners because stone tools are often much smaller than the recommended minimum field of view. Similarly, the resolution of conventional medical CT scanners might not be sufficient to capture minor details of stone tools, such as the outline or dorsal scars.

This protocol aims at providing the first detailed procedure dedicated to the scanning of small lithic implements for further three-dimensional analysis. Note that some of the steps must be repeated at different working stages throughout this protocol. In cases where a task must be done in the exact same way as described in a previous step, a reference to that step is provided. When slight changes were made, the step was modified and reported entirely. This

Citation: Dominik GÃÂ¶ldner, Fotios Alexandros Karakostis, Armando Falcucci StyroStone: A protocol for scanning and extracting three-dimensional meshes of stone artefacts using Micro-CT scanners https://dx.doi.org/10.17504/protocols.io.4r3l24d9qg1y/v2

This is an open access protocol distributed under the terms of the **Creative Commons Attribution License** (https://creativecommons.org/licenses/by/4.0/), which permits unrestricted use, distribution, and reproduction in any medium,

protocol contains a few red and green colours (e.g., arrows or within-program colours) which might be perceived differently by people with dyschromatopsia. However, the display of these colours has been kept to a minimum.

We recommend the reader to go over the entire protocol carefully, even if only some specific parts are required. A few points are in fact interdependent, and some of them must be applied simultaneously.

Content:
Part 1 – Styrofoam preparation
Part 2 – Micro-CT scanning
Part 3 – 3D model extraction of CT scanned stone artifacts using Avizo
Part 4 – Cropping extracted surface model to separate Face A and B in Artec Studio
Part 5 – Cropping Face A to separate the lines in Artec Studio
Part 6 – Cropping each stone artefact from the lines in Artec Studio
Part 7 – Virtually control measurements in MeshLab
Part 8 – Artec scanning of larger artifacts
Part 9 – Export meshes as non-binary ply models for successive analysis in *geomorph*

Three-dimensional example (in *ply* format) of the effectivity of the StyroStone Protocol:
You can download an example of one Styrofoam line in 3D obtained using our protocol to appreciate the result that can be achieved. We have selected a line where objects are characterized by different metric and morphological attributes. Notice the retouching well visible in the last five smaller artifacts (counting from the left when artifact are oriented with the dorsal face in front of the observer and the butt down), as well as the platforms and bulbs of all artifacts. The rest of the 3D meshes available on Zenodo (https://doi.org/10.5281/zenodo.6362150).
Example.ply

DOI

dx.doi.org/10.17504/protocols.io.4r3l24d9qg1y/v2

Dominik Göldner, Fotios Alexandros Karakostis, Armando Falcucci 2022. StyroStone: A protocol for scanning and extracting three-dimensional meshes of stone artefacts using Micro-CT scanners. **protocols.io**
https://dx.doi.org/10.17504/protocols.io.4r3l24d9qg1y/v2
Armando Falcucci

Deutsche Forschungsgemeinschaft (DFG)
Grant ID: 431809858 – FA 1707/1-1

lithics, geometric morphometrics, 3D scanning, CT, micro-CT, Artec, Artec Space Spider scanner, archaeology, Avizo, MeshLab, virtual, segmentation, stone tools, GM, GMM, Artec Studio Professional, 3D, three-dimensional

Citation: Dominik GÃÂ¶ldner, Fotios Alexandros Karakostis, Armando Falcucci StyroStone: A protocol for scanning and extracting three-dimensional meshes of stone artefacts using Micro-CT scanners https://dx.doi.org/10.17504/protocols.io.4r3l24d9qg1y/v2

This is an open access protocol distributed under the terms of the **Creative Commons Attribution License** (https://creativecommons.org/licenses/by/4.0/), which permits unrestricted use, distribution, and reproduction in any medium,

protocol ,

Mar 18, 2022

Mar 18, 2022

Mar 18, 2022 Dominik Göldner

59602

Sections often start with a short preface or introduction including useful background information, notes, or tips inserted in the first step right before the actually step.

Occasionally, notes and technical warnings, which are both indicated as such, are included in steps where necessary or useful.

Program options or commands are indicated with single quotes (e.g., 'XYZ').

Whenever referred to another step within a step, the reference step is highlighted in bold.

References

Adams, D., Collyer, M., Kaliontzopoulou, A., Baken, E., 2021. Geomorph: Software for geometric morphometric analyses. R package version 4.0. https://cran.r-project.org/package=geomorph

Archer, W., Djakovic, I., Brenet, M., et al., 2021. Quantifying differences in hominin flaking technologies with 3D shape analysis. Journal Of Human Evolution 150, 102912

Artec Studio Professional 13. User´s Guide / Manual. http://docs.artec-group.com/as/13/en
Cignoni, P., Callieri, M., Corsini, M., Dellepiane, M., Ganovelli, F., Ranzuglia, G., 2008. MeshLab: an Open-Source Mesh Processing Tool. Sixth Eurographics Italian Chapter Conference, 129-136

R Core Team (2020). R: A language and environment for statistical computing. R Foundation for Statistical Computing, Vienna, Austria. URL https://www.R-project.org/

Thermo Scientific™ Avizo™ Software 9 User’s Guide, Berlin. https://assets.thermofisher.com/TFS-Assets/MSD/Product-Guides/user-guide-avizo-software.pdf

Citation: Dominik GÃÂ¶ldner, Fotios Alexandros Karakostis, Armando Falcucci StyroStone: A protocol for scanning and extracting three-dimensional meshes of stone artefacts using Micro-CT scanners https://dx.doi.org/10.17504/protocols.io.4r3l24d9qg1y/v2

This is an open access protocol distributed under the terms of the **Creative Commons Attribution License** (https://creativecommons.org/licenses/by/4.0/), which permits unrestricted use, distribution, and reproduction in any medium,

micro-CT scanning:

- A micro-CT scanner
- Associated scanning software (e.g., Phoenix Datos-X software; General Electronics MCC).
- A workstation powerful enough to process large 3D datasets
  - Used here:
    - Operating system: Microsoft Windows 7 Enterprise, Version: 6.1.7601 Service Pack 1 Build 7601
    - System type: x64-based PC
    - Processor: Intel® Xeon® CPU E5-2630 v4, 2.20 GHz, 2201 Mhz, 10 Cores, 20 Logical Processors
    - Graphics card: Nvidia GeForce gtx1080

Surface scanning:

- High-resolution 3D surface scanner (e.g., see above; Artec Space Spider Scanner attendant software, i.e., Artec Studio Professional, version 13.2.3.59)
- Rotating turntable
- Professional modelling clay
- Anti-translucent spray

3D scanning and processing software:

- Artec Studio Professional Software (used version: v15.0.3.425)
- MeshLab (used version: v2016.12)
- Avizo Lite (Thermo Fisher Scientific; Waltham Massachusetts; used version: 9.2.0)
- Microsoft Office (i.e., Microsoft Excel)

Other materials:

- Digital camera
- Styrofoam
- Plastic rubber bands
- Box cutter

Part 1 - Styrofoam preparation

1 **Note:** Depending on the number of lithics that need to be scanned, this section might take several hours.

Select a blank Styrofoam body (or similar material) to hold the artefacts. It should be thick enough to carve holes on both sides (Face A and Face B).

2 Identify the scanning size limitations of the micro-CT scanner.

3 Cut the Styrofoam body into a rectangle according to the dimensions determined in **Step 2.** Our dimensions: 25 cm (height) x 22 cm (width) x 5 cm (thickness).

3.1 To increase the stability, you might add an additional Styrofoam support as base of ca. 2 cm height and a width of a few cm wider than the area of contact between the two parts (our total Styrofoam body height was 27 cm).

Citation: Dominik GÃÂ¶ldner, Fotios Alexandros Karakostis, Armando Falcucci StyroStone: A protocol for scanning and extracting three-dimensional meshes of stone artefacts using Micro-CT scanners https://dx.doi.org/10.17504/protocols.io.4r3l24d9qg1y/v2

This is an open access protocol distributed under the terms of the **Creative Commons Attribution License** (https://creativecommons.org/licenses/by/4.0/), which permits unrestricted use, distribution, and reproduction in any medium,

3.2 Glue the two Styrofoam pieces together with Polyvinyl acetate (white glue).

4 Draw guiding or separation lines with a dark marker on both sides (Face A and B) of the Styrofoam body depending on the size and number of artefacts that need to be scanned – these will determine the rows or lines in which the artefacts will be placed one after another.

The row separation will help to keep the artefacts in the same orientation and will simplify the subsequent 3D model extraction after the CT-scanning process.

5 Draw a mark (e.g., a dot or "A") on the first side of the Styrofoam (Face A), which will later facilitate the correct identification of the artefacts.

6 Carve holes along rows into the Styrofoam with a box cutter that are roughly the same dimensions of the artefacts. They should be secure enough to ensure a stable positioning and protect the artefacts from moving during the CT-scanning process.

6.1 Insert the artefact directly into its hole and immediately document its position in the Styrofoam as described in **Step 6.2** (Fig. 1).

On Face A, place the first artefact on the left most position of the first row and continue in this line towards the right until the line is fully occupied. Then continue with the second row below, again starting at the left side until Face A is completed. Continue with Face B in the same way.

Insert lithics following the best-suited criteria depending on how the lithic assemblages are curated (e.g., stratigraphic units, raw materials, ID numbers) or according to the size variation of the artefacts if necessary.

Always use the same orientation of the artefacts, i.e., dorsal face facing upwards and proximal side at the bottom. By placing the longest axis of the specimen vertically, the object thickness between the source and the detector remains limited during the scanning process, requiring less x-ray penetration power and thus reducing energy costs. Moreover, during the later post-processing step, such an orientation will enable easier identification and virtual separation among 3D scans of lithics when compared with the reference list of artefacts (**Step 6.2**) and reference photographs of the Styrofoam body (**Step 7.1 and 7.2**).

In our case, we scanned ca. 700 lithics with the CT-scanner using 4 Styrofoam bodies each with two faces (A and B) for a total of 8 faces. Each face consisted of ca. 4 to 8 lines and each line contained ca. 10 to 20 artefacts (total of ca. 60 to 120 artefacts per face).

Citation: Dominik GÃÂ¶ldner, Fotios Alexandros Karakostis, Armando Falcucci StyroStone: A protocol for scanning and extracting three-dimensional meshes of stone artefacts using Micro-CT scanners https://dx.doi.org/10.17504/protocols.io.4r3l24d9qg1y/v2

This is an open access protocol distributed under the terms of the **Creative Commons Attribution License** (https://creativecommons.org/licenses/by/4.0/), which permits unrestricted use, distribution, and reproduction in any medium,

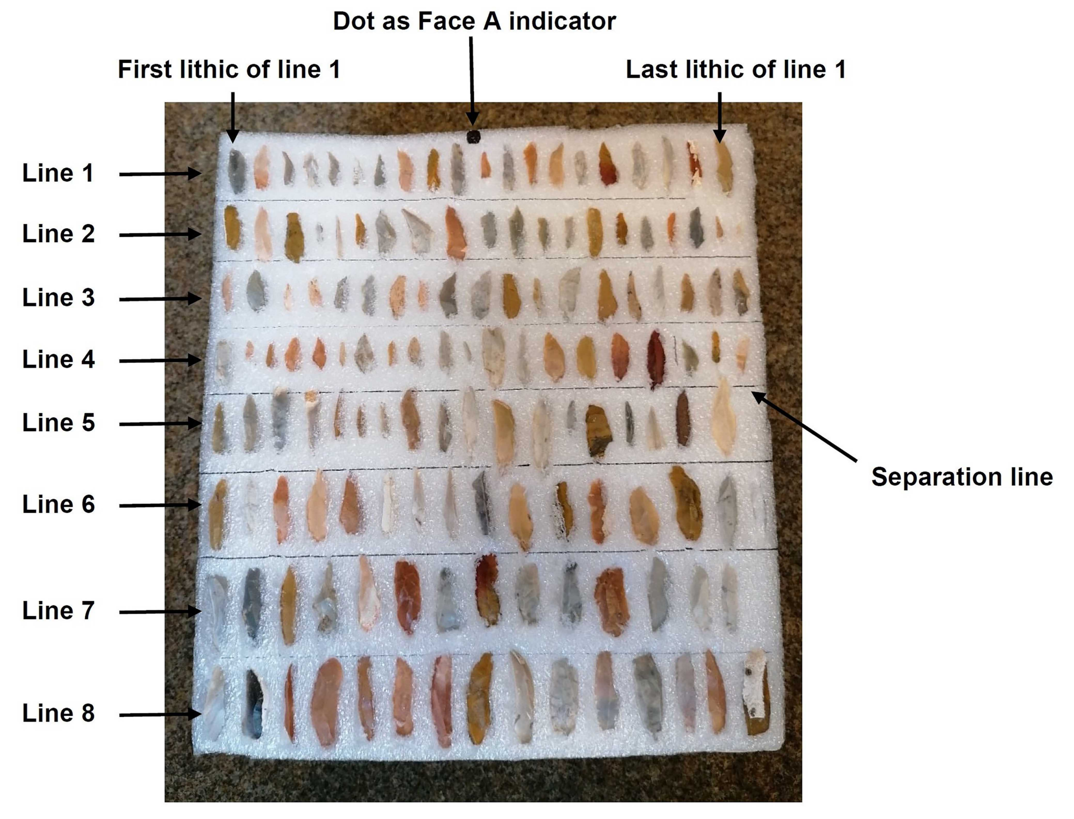

Fig. 1: Overview of a completely filled Styrofoam body (Face A).

6.2 Document the exact position of each artefact in a spreadsheet (e.g., MS Excel) directly after it is inserted into its Styrofoam hole.

Clearly indicate the Styrofoam side, row, and at which numeric position the artefacts are located. For simplicity reasons you might use a coded identification key.

Example: An artefact with the location code **S1-FA-L2.5** would be inserted into first Styrofoam body (***S1***; only in cases where several bodies must be used to scan large amounts of lithics) Face A (***FA***, first Styrofoam side) in the second row from the top to the bottom (***L2***) and at the fifth position from the left to the right (***.5***) in this row.

Keeping track of the positions is extremely important and will not only ensure the fast and correct labelling of the right artefact ID with the 3D models, but also ensure the artefacts are safely returned to their find bags. Therefore, you might use a handwritten list as backup.

7 Take reference pictures.

7.1 After Face A and B are filled, take reference pictures of both faces.

Citation: Dominik GÃÂ¶ldner, Fotios Alexandros Karakostis, Armando Falcucci StyroStone: A protocol for scanning and extracting three-dimensional meshes of stone artefacts using Micro-CT scanners https://dx.doi.org/10.17504/protocols.io.4r3l24d9qg1y/v2

This is an open access protocol distributed under the terms of the **Creative Commons Attribution License** (https://creativecommons.org/licenses/by/4.0/), which permits unrestricted use, distribution, and reproduction in any medium,

7.2 Take a picture of the first artefact of the first row of each face. This will serve as a reference to orient the 3D model of the entire face, making the later identification of the artefacts' order much easier.

8 Cover the Styrofoam faces with Styrofoam slabs.

8.1 Cover both Faces A and B with a thin Styrofoam slab (ca. 1 cm thickness) of the same dimensions as the main Styrofoam body (**Step 3**).

8.2 Tightly secure the two Styrofoam slabs and main Styrofoam body with plastic rubber bands (Fig. 2). Do not glue them together, as it will be difficult to disassemble when removing the lithics after scanning.

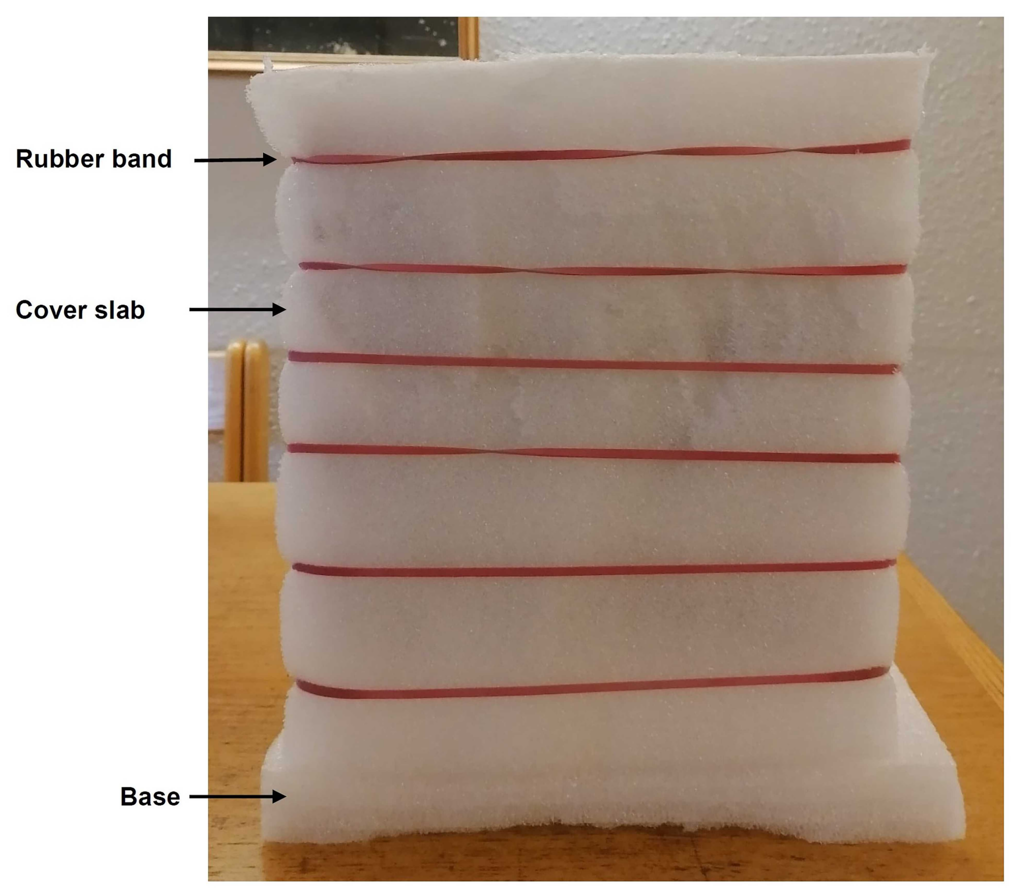

Fig. 2: Sealed Styrofoam bodies (cover slabs and main body) on its base and held together with rubber bands.

Citation: Dominik GÃÂ¶ldner, Fotios Alexandros Karakostis, Armando Falcucci StyroStone: A protocol for scanning and extracting three-dimensional meshes of stone artefacts using Micro-CT scanners https://dx.doi.org/10.17504/protocols.io.4r3l24d9qg1y/v2

This is an open access protocol distributed under the terms of the **Creative Commons Attribution License** (https://creativecommons.org/licenses/by/4.0/), which permits unrestricted use, distribution, and reproduction in any medium,

9 Repeat **Step 1 to Step 8.2** if more Styrofoam bodies are needed (S1, S2, etc.; see **Step 6.2** for labelling).

9.1 Label each Styrofoam body with a number to identify them afterwards (Fig. 3).

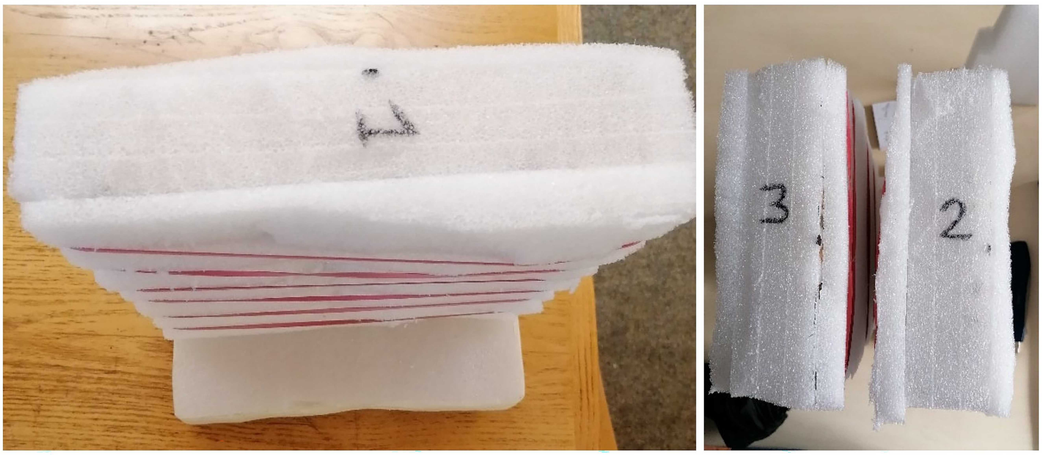

Fig. 3: Styrofoam bodies labelled with “1”, “2” and “3” for identification. The black dots indicate the position of Face A.

Part 2 - Micro-CT scanning

10 **Note:** CT-scanning is usually performed by a certified and trained technician using the scanning parameters specified below and depending on the inherent nature of the different raw materials. The following steps are a summary of the scanning process performed for our case study, which relied on the scanning of fine-grained chert bladelets with thicknesses between 0.5 mm to 6.0 mm (mean: 2.4 mm).

In our study, each scan took approximately 2:20 hours and we scanned up to 220 bladelets per scan (these bladelets had an average size of 28 x 8 x 2.4 mm).

Place the Styrofoam body on the turning base of the CT-scanner (Fig. 4).

Citation: Dominik GÃÂ¶ldner, Fotios Alexandros Karakostis, Armando Falcucci StyroStone: A protocol for scanning and extracting three-dimensional meshes of stone artefacts using Micro-CT scanners https://dx.doi.org/10.17504/protocols.io.4r3l24d9qg1y/v2

This is an open access protocol distributed under the terms of the **Creative Commons Attribution License** (https://creativecommons.org/licenses/by/4.0/), which permits unrestricted use, distribution, and reproduction in any medium,

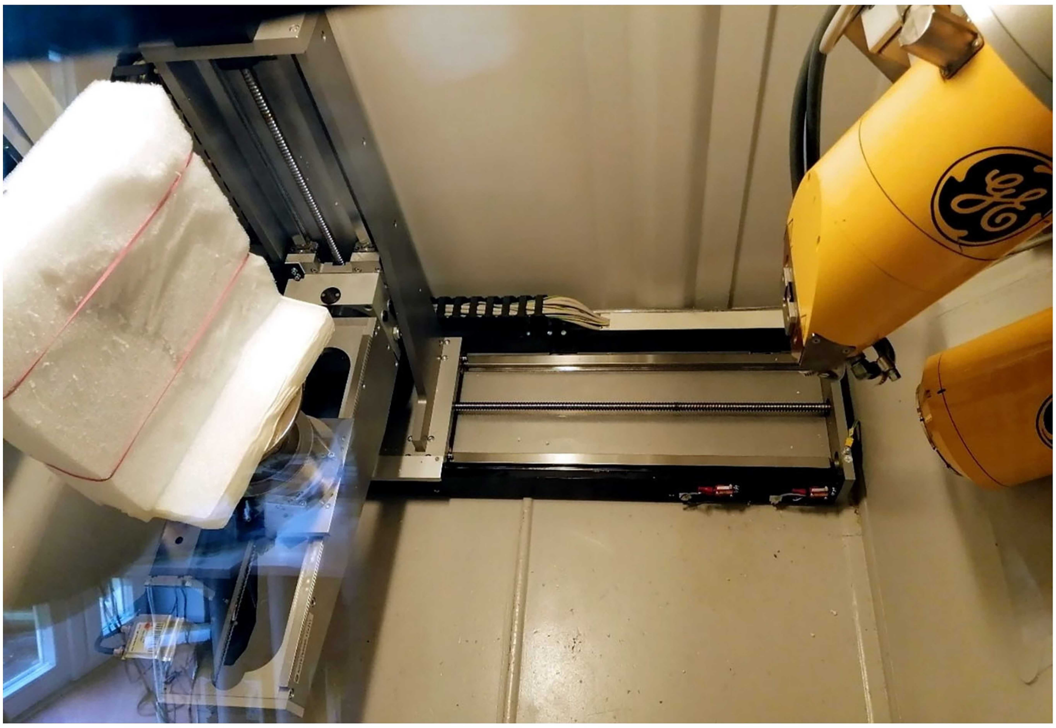

Fig. 4: Styrofoam body placed and secured on the turning base in the micro-CT scanner.

11 Secure it with a strong double-sided tape on the base.

12 The turning base should be positioned as far as possible from the source to increase the field of view and, therefore, the number of lithics that can be scanned at one time. Note that with greater distance from the source, the resolution decreases.

13 Set up the machine according to the best scanning parameters – usually done by the scanning technician.

For scanning multiple specimens at the same time, it is advisable to use the "multiscans" option, which enables the device to scan longer targets within the same scanning session. In particular, using the scanner's associated computer device and software (e.g., Phoenix Datos-X software; General Electronics MCC), the scanning technician virtually separates the target into different layers (by Styrofoam height) to be scanned individually (per layer/scan), which are then automatically merged before model reconstruction. It must be highlighted that the use of this option might produce visual artefacts in the resulting 3D models, appearing as lines at the borders in between the layered scans (i.e., "merging lines"). To ensure that these lines will not affect stone tool morphology, the technician should make sure that the selected upper and lower borderlines of each layer/scan do not intersect any stone tools.

The resolution used across our scans was ca. 140 microns. Resolution largely depends on the distance

Citation: Dominik GÃÂ¶ldner, Fotios Alexandros Karakostis, Armando Falcucci StyroStone: A protocol for scanning and extracting three-dimensional meshes of stone artefacts using Micro-CT scanners https://dx.doi.org/10.17504/protocols.io.4r3l24d9qg1y/v2

This is an open access protocol distributed under the terms of the **Creative Commons Attribution License** (https://creativecommons.org/licenses/by/4.0/), which permits unrestricted use, distribution, and reproduction in any medium,

between the source and the target/object. Even though the utilized micro-CT scanner is technically capable of producing models with much greater resolution, our experiments showed that 140 microns was a good balance, allowing us to scan a large number of artefacts at once, while avoiding distinguishable inaccuracies in the resulting 3D surface form (size and shape) of the blades and bladelets.

14 Start scanning process.

15 After the scanning process is completed, save the scans onto a hard disk. Make sure to save the scanning metadata (e.g., as .pcr file), including all scanning parameters, which are required to open and scale the CT scans (e.g., in Avizo). Important scanning parameters to successfully load the CT data into Avizo Lite are the resolution (Voxel size), volume dimensions (x, y, z), and image data type (e.g., 16 bit).

16 Visualize the overall scanning result to check for obvious errors. This can be done by reconstructing the CT data and exporting the file as an image stack, e.g., as .tiff files or .vol files, which can be opened in Avizo Lite. Reconstruction of the CT data can be performed, visualized, and exported using software like Phoenix Datos-X.

17 Take the scanned Styrofoam body from the CT scanner.

18 Dismantle the Styrofoam body by taking off the plastic rubber strings and the covering plates.

19 Remove the artefacts one by one from the Styrofoam and put them back into their respective find bags.

Part 3 – 3D model extraction of CT scanned stone artefacts using Avizo

20 **Note:** For our project the preparation and separation of the faces took around 2 hours per Styrofoam body. The subsequent extraction of each lithic described in the next chapter (Part/Section 4) only took around one minute per artefact.

Citation: Dominik GÃÂ¶ldner, Fotios Alexandros Karakostis, Armando Falcucci StyroStone: A protocol for scanning and extracting three-dimensional meshes of stone artefacts using Micro-CT scanners https://dx.doi.org/10.17504/protocols.io.4r3l24d9qg1y/v2

This is an open access protocol distributed under the terms of the **Creative Commons Attribution License** (https://creativecommons.org/licenses/by/4.0/), which permits unrestricted use, distribution, and reproduction in any medium,

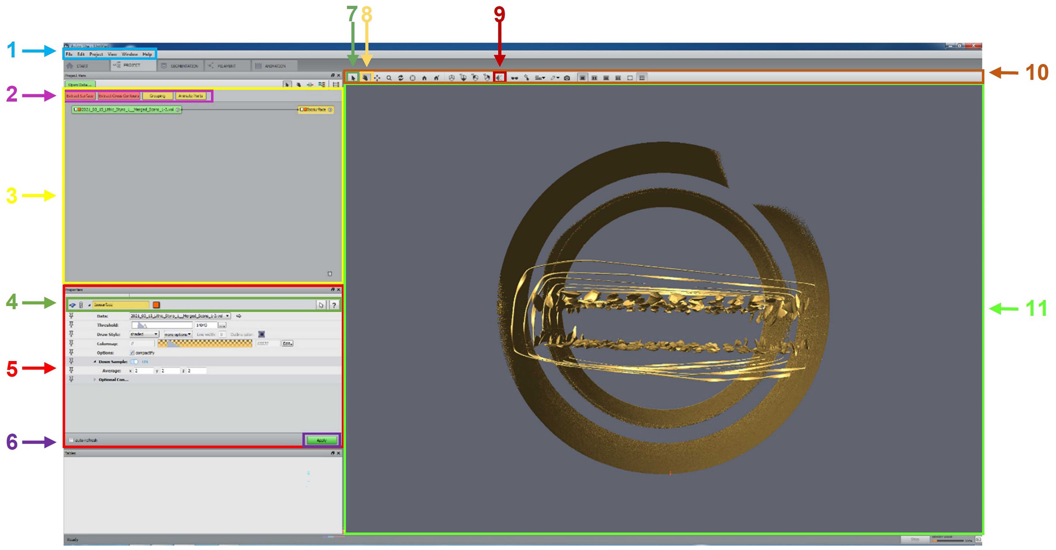

Fig. 5: Program overview of the most important tools and panels used in Avizo.

Legend:

1 (Main) Menu Bar
2 Options Menu Bar of the Label Panel
3 Label Panel
4 Options Menu Bar of the Properties Panel
5 Properties Panel
6 Apply Button in the Properties Panel to set new properties to labels
7 Courser Tool to select points etc. in the 3D View Panel
8 Dragging Tool to move the 3D model in the 3D View Panel
9 Orthographic View
10 Option Menu Bar for the 3D View Panel
11 3D View Panel

Open CT scan in Avizo Lite.

20.1 Open Avizo and load CT scan (.vol file).

20.2 In ‘File Format Selection’ choose ‘Raw Data’ and press ‘Okay’ (Fig. 6).

Citation: Dominik GÃÂ¶ldner, Fotios Alexandros Karakostis, Armando Falcucci StyroStone: A protocol for scanning and extracting three-dimensional meshes of stone artefacts using Micro-CT scanners https://dx.doi.org/10.17504/protocols.io.4r3l24d9qg1y/v2

This is an open access protocol distributed under the terms of the **Creative Commons Attribution License** (https://creativecommons.org/licenses/by/4.0/), which permits unrestricted use, distribution, and reproduction in any medium,

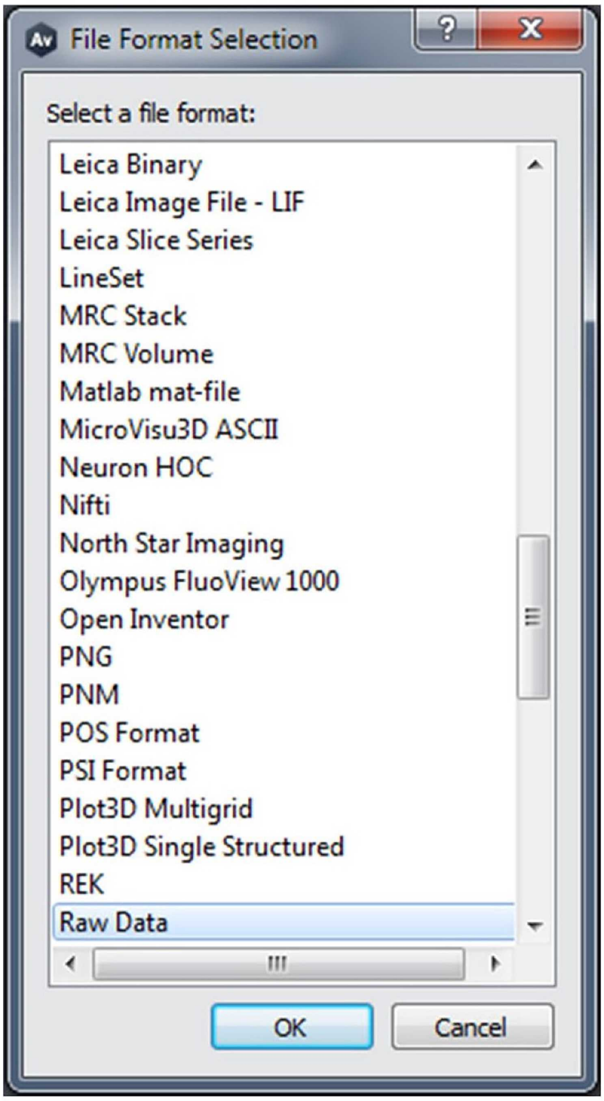

Fig. 6: 'File Format Selection' window.

20.3 In 'Out-of-Core Data' choose 'Read complete volume into memory' and press 'Okay' (Fig. 7).

Citation: Dominik GÃÂ¶ldner, Fotios Alexandros Karakostis, Armando Falcucci StyroStone: A protocol for scanning and extracting three-dimensional meshes of stone artefacts using Micro-CT scanners https://dx.doi.org/10.17504/protocols.io.4r3l24d9qg1y/v2

This is an open access protocol distributed under the terms of the **Creative Commons Attribution License** (https://creativecommons.org/licenses/by/4.0/), which permits unrestricted use, distribution, and reproduction in any medium,

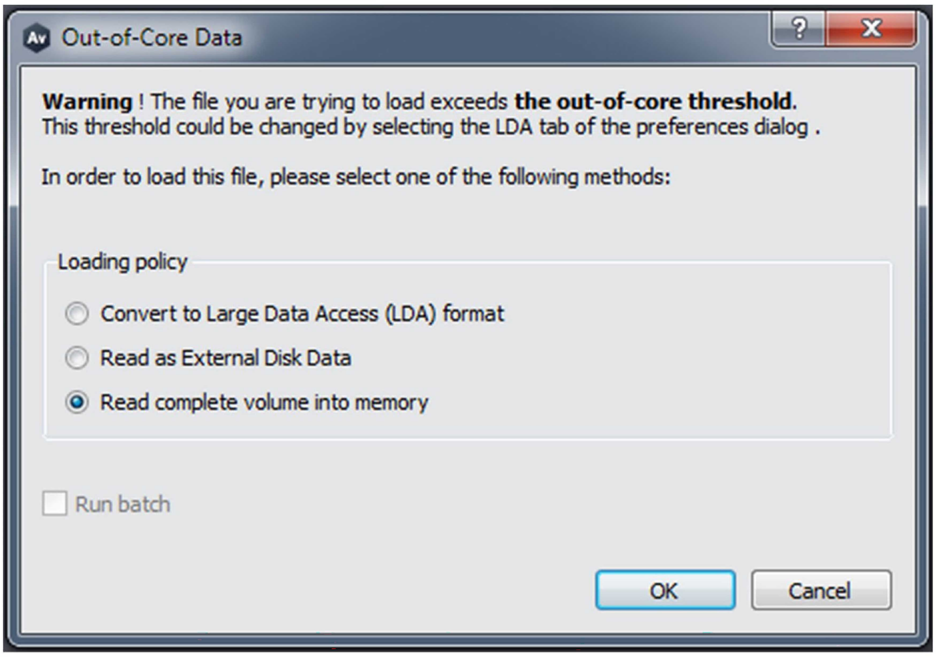

Fig. 7: 'Out-of-Core Data' window.

20.4 Indicate raw data parameters in 'Raw Data Parameters' window (Fig. 8).

20.5 Set 'Data type' according to scanner settings (usually '16-bit unsigned') (blue arrow in Fig. 8), leave the rest as the pre-set values.

Citation: Dominik GÃÂ¶ldner, Fotios Alexandros Karakostis, Armando Falcucci StyroStone: A protocol for scanning and extracting three-dimensional meshes of stone artefacts using Micro-CT scanners https://dx.doi.org/10.17504/protocols.io.4r3l24d9qg1y/v2

This is an open access protocol distributed under the terms of the **Creative Commons Attribution License** (https://creativecommons.org/licenses/by/4.0/), which permits unrestricted use, distribution, and reproduction in any medium,

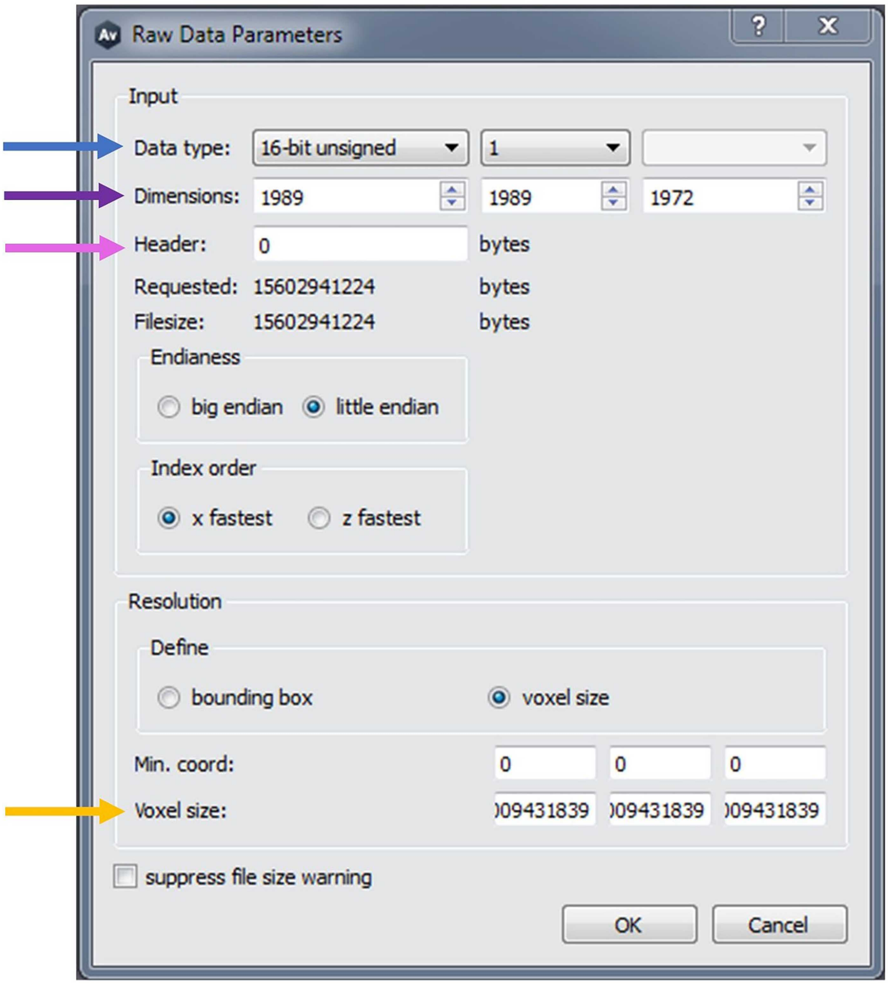

Fig. 8: 'Raw Data Parameters' window.

20.6 Open CT meta-data file (e.g., pcr in this study). It should open automatically in notepad (Fig. 9).

Citation: Dominik GÃÂ¶ldner, Fotios Alexandros Karakostis, Armando Falcucci StyroStone: A protocol for scanning and extracting three-dimensional meshes of stone artefacts using Micro-CT scanners https://dx.doi.org/10.17504/protocols.io.4r3l24d9qg1y/v2

This is an open access protocol distributed under the terms of the **Creative Commons Attribution License** (https://creativecommons.org/licenses/by/4.0/), which permits unrestricted use, distribution, and reproduction in any medium,

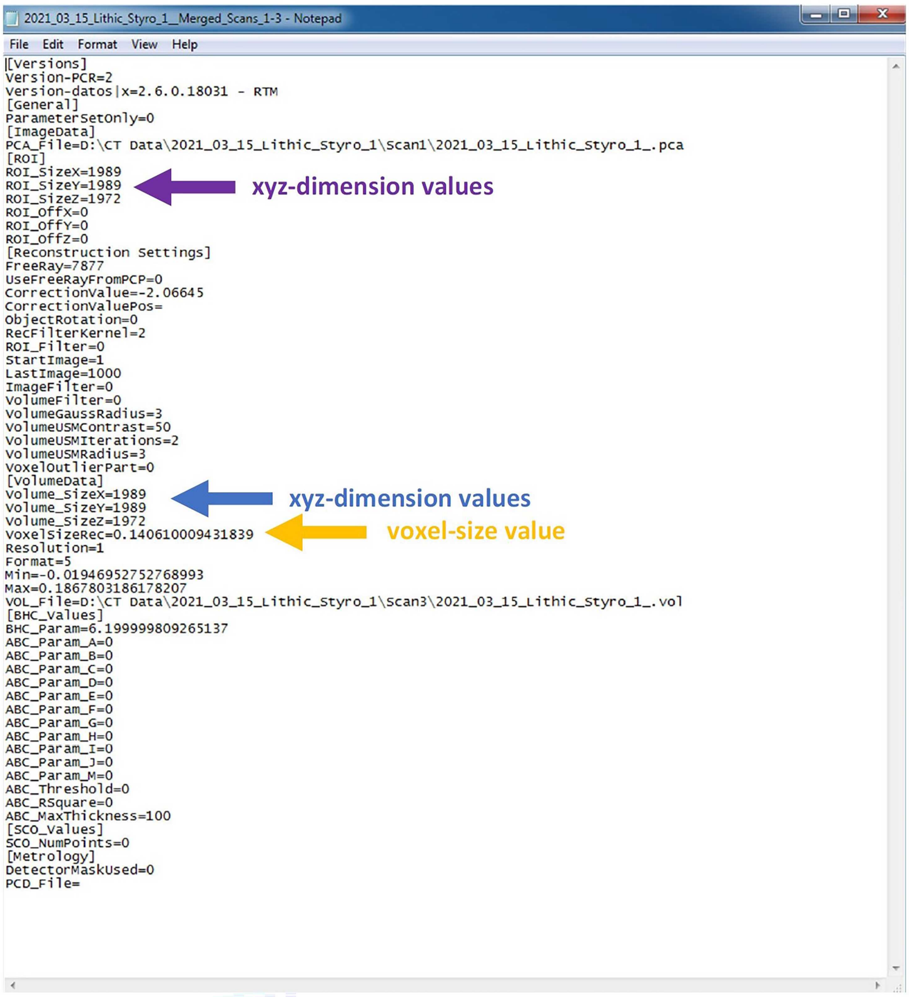

Fig. 9: PCR file with CT scan metadata opened in Notepad.

20.7 To enter the x-, y-, and z-dimensions in the 'Raw Data parameters' window (violet arrow in Fig. 8) search for 'ROI_SizeX=NUMBER', 'ROI_SizeY=NUMBER' and 'ROI_SizeZ=NUMBER' respectively (violet arrow in Fig. 9).

Note: you should usually aim for the parameters 'Volume_Size[X, Y, or Z]' (blue arrow in Fig. 9), especially when the ROI (Region of Interest) was specified before the scanning process. In our case ROI was not specified and is therefore identical to the volume size. If ROI is specified by the technician, the ROI parameter reported in the metadata file will only show a part of the image.

Caution: 'Header' value (pink arrow in Fig. 8) in 'Raw Data Parameters' window will change to 0 if all dimensions are entered correctly.

20.8 To enter the voxel size value into 'Raw Data parameters' window (yellow arrow in Fig. 8), search for 'VoxelSizeRec=NUMBER' (yellow arrow in Fig. 9).

Citation: Dominik GÃÂ¶ldner, Fotios Alexandros Karakostis, Armando Falcucci StyroStone: A protocol for scanning and extracting three-dimensional meshes of stone artefacts using Micro-CT scanners https://dx.doi.org/10.17504/protocols.io.4r3l24d9qg1y/v2

This is an open access protocol distributed under the terms of the **Creative Commons Attribution License** (https://creativecommons.org/licenses/by/4.0/), which permits unrestricted use, distribution, and reproduction in any medium,

20.9 Leave all other parameters in 'Raw Data Parameters' window as the pre-sets and press 'Okay'.

20.10 Indicate unit in 'Units Editor' window (Fig. 10) according to indicated voxel size unit in pcr CT-scan metadata file ('VoxelSizeRec=NUMBER', yellow arrow in Fig. 9) and press 'Okay'.

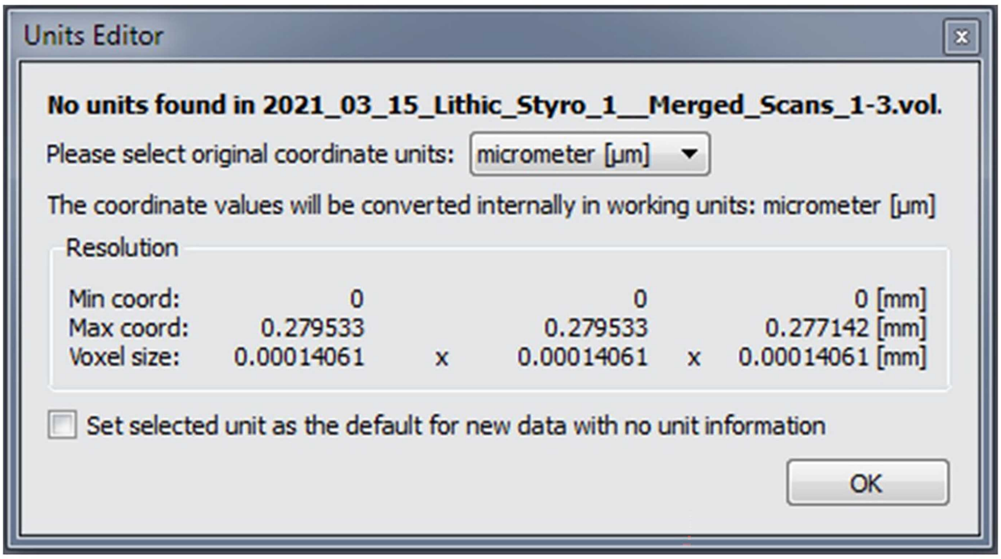

Fig. 10: 'Units Editor window'.

20.11 Now, Avizo should load and open the vol file (Fig. 11).

Citation: Dominik GÃÂ¶ldner, Fotios Alexandros Karakostis, Armando Falcucci StyroStone: A protocol for scanning and extracting three-dimensional meshes of stone artefacts using Micro-CT scanners https://dx.doi.org/10.17504/protocols.io.4r3l24d9qg1y/v2

This is an open access protocol distributed under the terms of the **Creative Commons Attribution License** (https://creativecommons.org/licenses/by/4.0/), which permits unrestricted use, distribution, and reproduction in any medium,

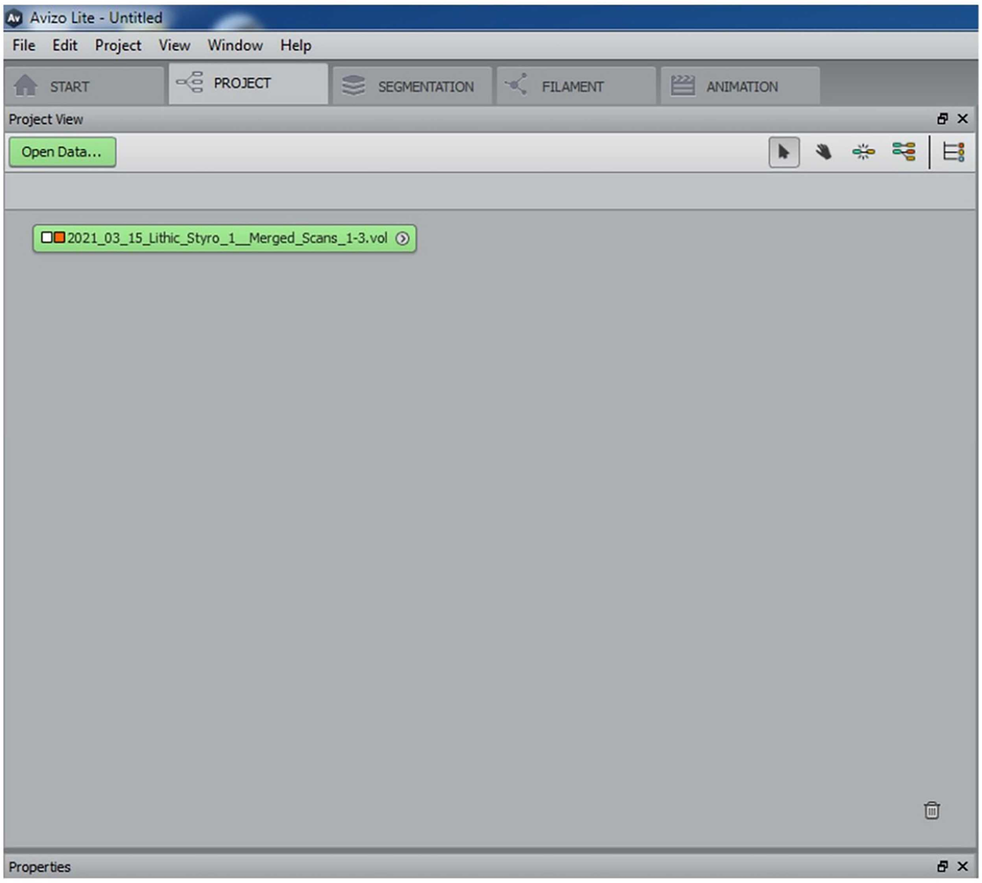

Fig. 11: Partial screenshot of the 'Label Panel' with loaded vol file.

21 Generate isosurface (also see manual: https://assets.thermofisher.com/TFS-Assets/MSD/Product-Guides/user-guide-avizo-software.pdf).

21.1 Click on vol file label and then on the 'Isosurface' button that appears in the options bar above to generate an isosurface of the scan. Alternatively, if it does not appear, click on the arrow sign at the right most position of the vol file label and enter 'Isosurface' in the search bar.

21.2 Click on the newly generated 'Isosurface' label (Fig. 12) and choose a 'Threshold' value (yellow arrow in Fig. 12) that separates the artefacts from the surrounding air, Styrofoam etc.

Enable and use 'Down Sample' (pink arrow in Fig. 12) to run a fast isosurface test extraction to check whether the chosen 'Threshold' value works well.

Time can be saved by finding a good threshold, as this lessens the need for cleaning and outlier removal. Note that a preview mode is not available, so it is not possible to judge whether the chosen 'Threshold' value works well overall. Therefore, it might be necessary to test multiple values before a good separation can be achieved (**Step**

Citation: Dominik GÃÂ¶ldner, Fotios Alexandros Karakostis, Armando Falcucci StyroStone: A protocol for scanning and extracting three-dimensional meshes of stone artefacts using Micro-CT scanners https://dx.doi.org/10.17504/protocols.io.4r3l24d9qg1y/v2

This is an open access protocol distributed under the terms of the **Creative Commons Attribution License** (https://creativecommons.org/licenses/by/4.0/), which permits unrestricted use, distribution, and reproduction in any medium,

21.5). It might be useful to note down the values that have already been tested and to narrow down a suitable Threshold value range.

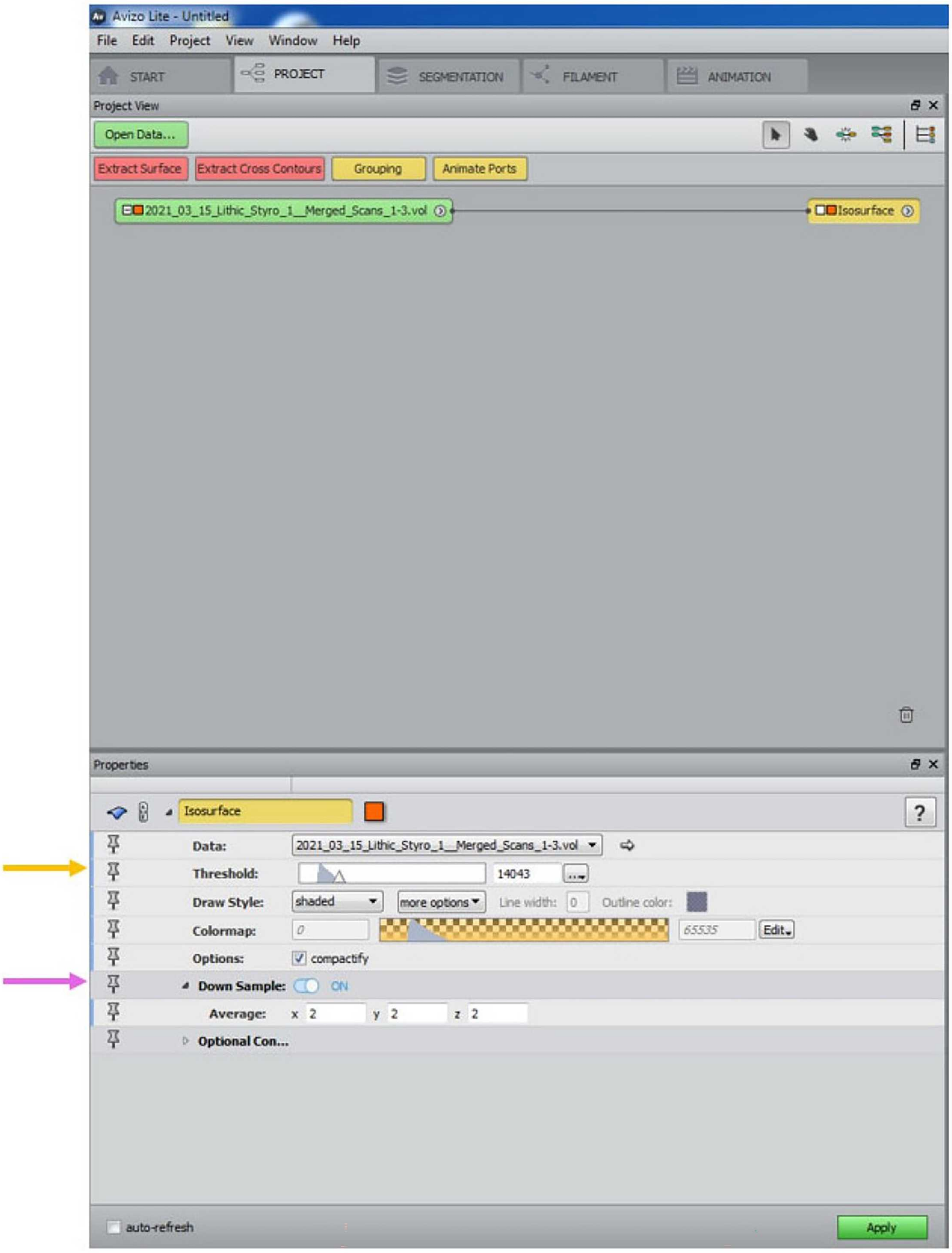

Fig. 12: Project view with generated 'Isosurface' label. Parameters for the isosurface can be adjusted in the 'Properties' panel below.

21.3 Press 'Apply' and wait until the process completed.

21.4 When completed, inspect the resulting isosurface model (Fig. 13 and 14) and check for

Citation: Dominik GÃÂ¶ldner, Fotios Alexandros Karakostis, Armando Falcucci StyroStone: A protocol for scanning and extracting three-dimensional meshes of stone artefacts using Micro-CT scanners https://dx.doi.org/10.17504/protocols.io.4r3l24d9qg1y/v2

This is an open access protocol distributed under the terms of the **Creative Commons Attribution License** (https://creativecommons.org/licenses/by/4.0/), which permits unrestricted use, distribution, and reproduction in any medium,

‘unusual’ or ‘unnatural’ surface inconstancies, ‘noise’, and scanning artefacts.

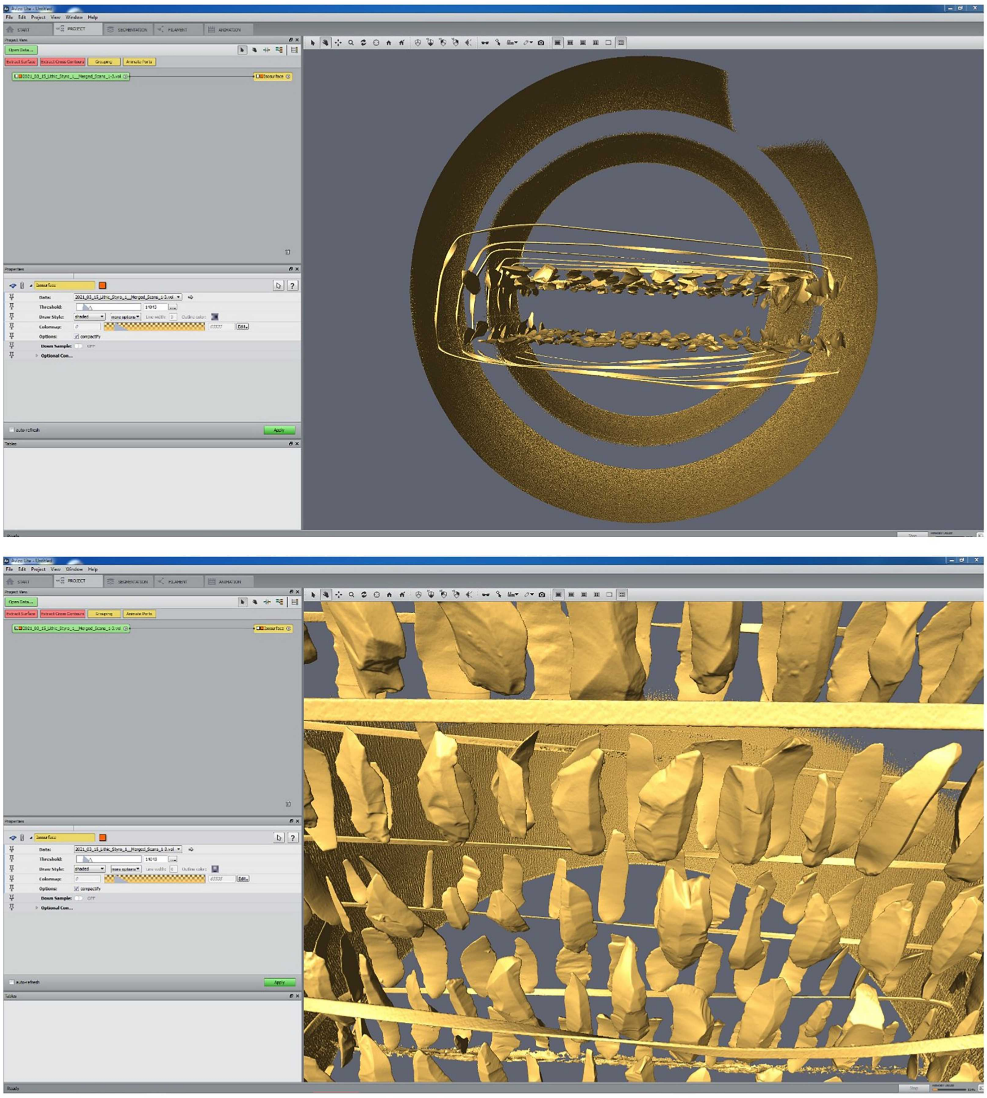

Fig. 13 (top) and 14 (bottom): Isosurface with clear separation between the lithic artefacts and other materials (e.g., rubber bands and Styrofoam).

21.5 If the chosen ‘Threshold’ value does not work well, repeat **Step 21.2** and **21.3** until a good separation is be achieved. If the chosen threshold works well, continue with **Step 21.6.**

21.6 If a suitable threshold value was found, repeat **Step 21.2** and **21.3** using the indicated threshold value (e.g., by copy and paste) but this time with ‘Down Sample’ disabled.

21.7 When completed, again inspect the resulting isosurface model (Fig. 13 and 14) and check surface inconstancies (see **Step 21.5**).

Citation: Dominik GÃÂ¶ldner, Fotios Alexandros Karakostis, Armando Falcucci StyroStone: A protocol for scanning and extracting three-dimensional meshes of stone artefacts using Micro-CT scanners https://dx.doi.org/10.17504/protocols.io.4r3l24d9qg1y/v2

This is an open access protocol distributed under the terms of the **Creative Commons Attribution License** (https://creativecommons.org/licenses/by/4.0/), which permits unrestricted use, distribution, and reproduction in any medium,

21.8 If the chosen ‘Threshold’ value does not work well, repeat **Step 21.2)** and **21.3** until a good separation is achieved.

22 Remove remaining materials.

22.1 Select the ‘Eye’ sign in the top menu bar above the 3D viewing panel (grey arrow in Fig. 15) to switch to orthographic view (‘Eye’ sign with parallel sight lines).

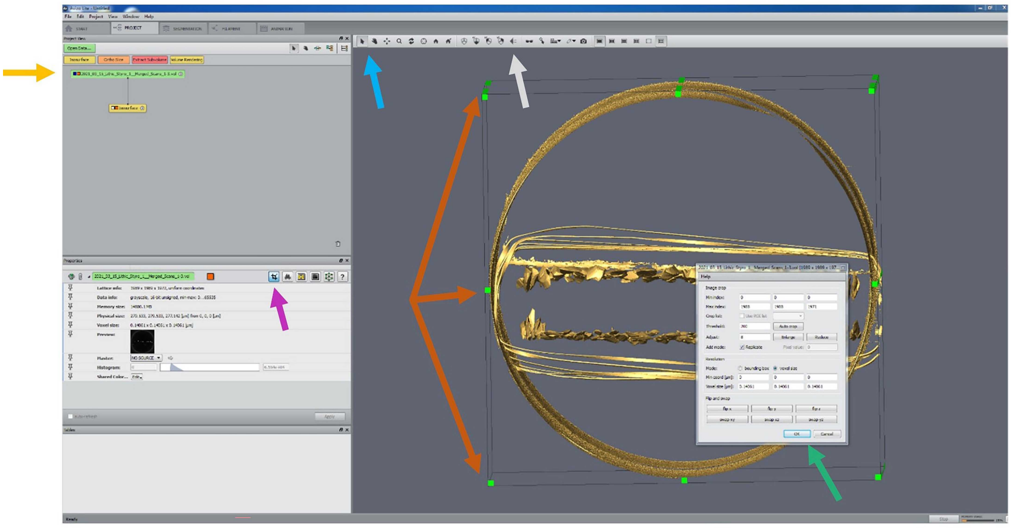

Fig. 15: Cropping process to remove outlying materials.

22.2 Select the vol file label (yellow arrow in Fig. 15).

22.3 Rotate the 3D model into a proper position (e.g., as shown in Fig. 15) that allows for easier removal of the unnecessary outlying material.

22.4 Select the ‘Cursor’ sign in the menu bar above the 3D viewing panel (blue arrow in Fig. 15).

22.5 Select the first ‘Tool’ sign (Cropping tool) in the options bar (pink arrow in Fig. 15).

Use the left mouse button and cursor to narrow the bounding box (brown arrows in Fig.

Citation: Dominik GÃÂ¶ldner, Fotios Alexandros Karakostis, Armando Falcucci StyroStone: A protocol for scanning and extracting three-dimensional meshes of stone artefacts using Micro-CT scanners https://dx.doi.org/10.17504/protocols.io.4r3l24d9qg1y/v2

This is an open access protocol distributed under the terms of the **Creative Commons Attribution License** (https://creativecommons.org/licenses/by/4.0/), which permits unrestricted use, distribution, and reproduction in any medium,

22.6 15). If possible, leave only the lithic artefacts within the bounding box (Fig. 16). Any remaining materials can still be excluded later if necessary. Switch between 'Cursor' and 'Hand' (i.e., dragging) mode and change the position of the 3D model to adjust bounding box in all dimensions to crop out as much of the outlying material as possible.

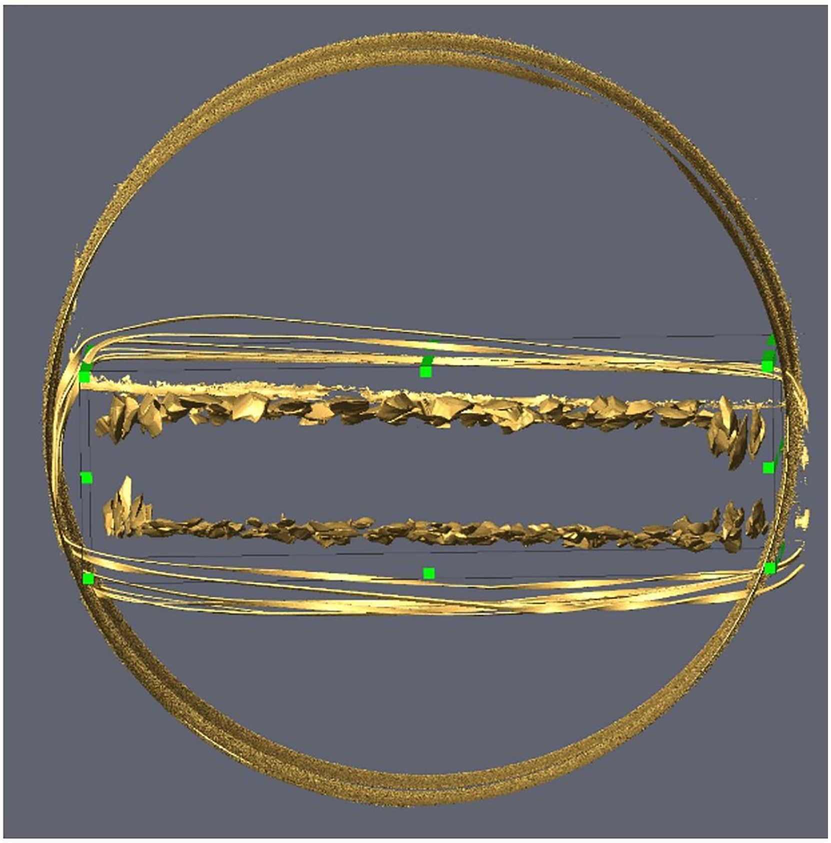

Fig. 16: Adjusted bounding box used to crop the outlying materials.

22.7 Apply changes to delete excluded materials with 'Ok' in cropping window (green arrow in Fig. 15).

23 Generate isosurface again for cropped scan.

23.1 Click on vol file label and then on the 'Isosurface' button (blue arrow in Fig. 17) that appears over the options bar to generate an isosurface of the scan (like **Step 21.1**).

Citation: Dominik GÃÂ¶ldner, Fotios Alexandros Karakostis, Armando Falcucci StyroStone: A protocol for scanning and extracting three-dimensional meshes of stone artefacts using Micro-CT scanners https://dx.doi.org/10.17504/protocols.io.4r3l24d9qg1y/v2

This is an open access protocol distributed under the terms of the **Creative Commons Attribution License** (https://creativecommons.org/licenses/by/4.0/), which permits unrestricted use, distribution, and reproduction in any medium,

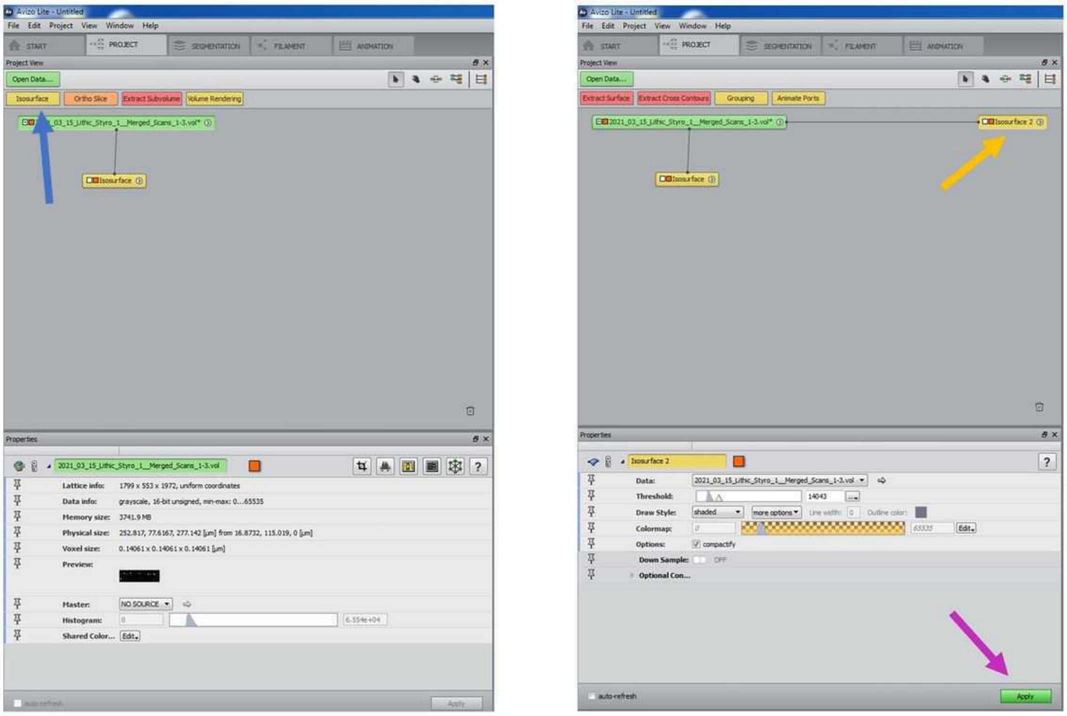

Fig. 17 (left) and 18 (right): Generation of cropped isosurface label.

23.2 Click on the newly generated ‘Ie,sosurface’ label (yellow arrow in Fig. 18). Disable ‘Down Sample’ and insert the ‘Threshold’ value identified to be sufficient earlier in **Step 21.2** or **Step 21.5 / 21.6**.

23.3 Click ‘Apply’ (pink arrow in fig. Fig. 18) and wait until the process has completed.

24 Extract surface from cropped isosurface.

24.1 Click on the newly generated isosurface (here ‘Isosurface 2’, blue arrow in Fig. 19).

Citation: Dominik GÃÂ¶ldner, Fotios Alexandros Karakostis, Armando Falcucci StyroStone: A protocol for scanning and extracting three-dimensional meshes of stone artefacts using Micro-CT scanners https://dx.doi.org/10.17504/protocols.io.4r3l24d9qg1y/v2

This is an open access protocol distributed under the terms of the **Creative Commons Attribution License** (https://creativecommons.org/licenses/by/4.0/), which permits unrestricted use, distribution, and reproduction in any medium,

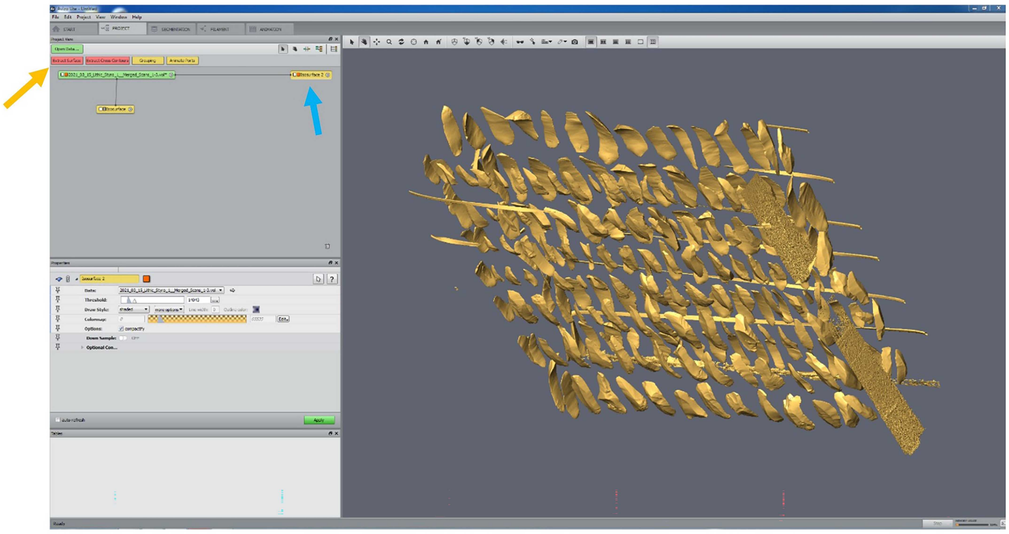

Fig. 19: Extracted surface model of all scanned stone artefacts. Some of the outlying material is still present and can be removed later.

24.2 Click ‘Extract Surface’ in the options bar above (yellow arrow in Fig. 19).

24.3 Select the newly created label called ‘Extract Surface’ (orange arrow in Fig. 20).

Citation: Dominik GÃÂ¶ldner, Fotios Alexandros Karakostis, Armando Falcucci StyroStone: A protocol for scanning and extracting three-dimensional meshes of stone artefacts using Micro-CT scanners https://dx.doi.org/10.17504/protocols.io.4r3l24d9qg1y/v2

This is an open access protocol distributed under the terms of the **Creative Commons Attribution License** (https://creativecommons.org/licenses/by/4.0/), which permits unrestricted use, distribution, and reproduction in any medium,

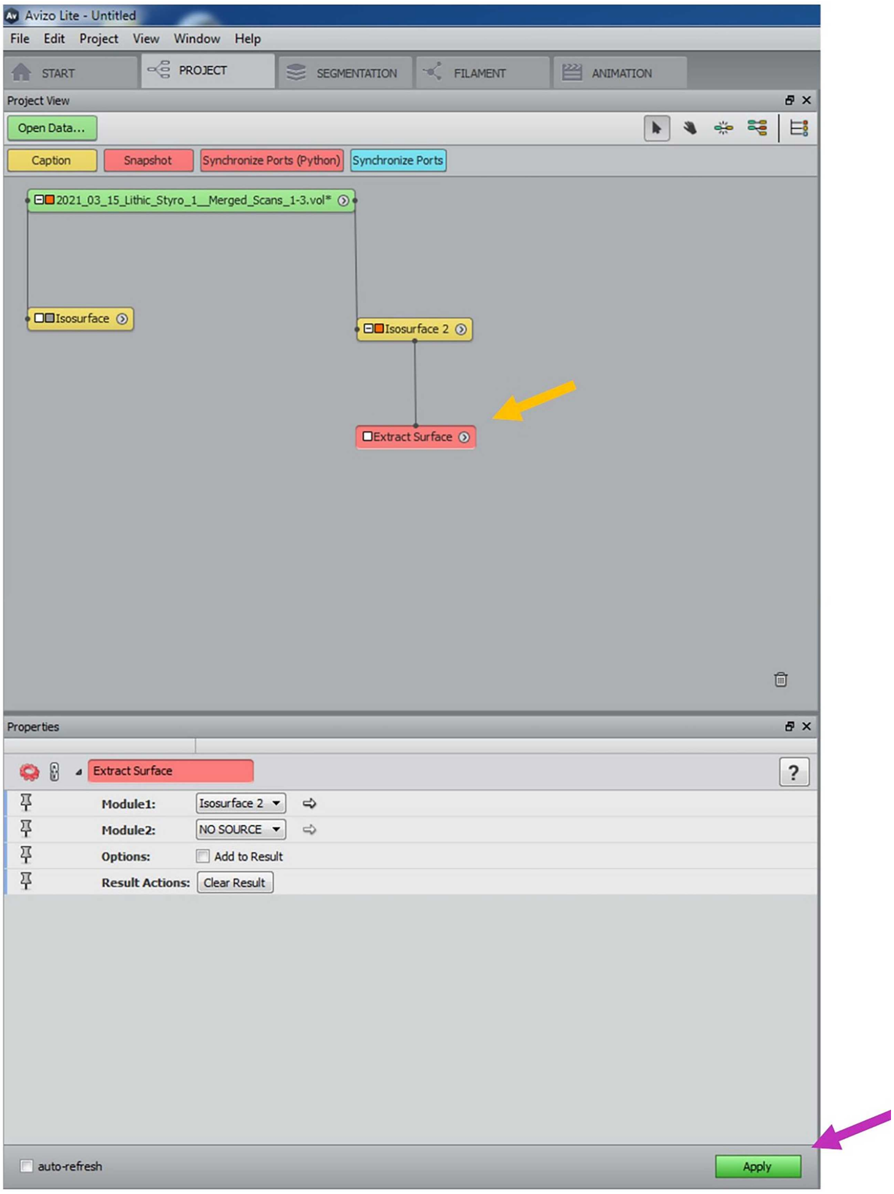

Fig. 20: Newly created 'Extract Surface' label from Isosurface.

24.4 Leave all parameters in the 'Extract Surface' menu as is and click 'Apply' to generate extracted surface (pink arrow in Fig. 20).

24.5 A new label called 'ExtractedSurface' will appear (blue arrow in Fig. 21).

Citation: Dominik GÃÂ¶ldner, Fotios Alexandros Karakostis, Armando Falcucci StyroStone: A protocol for scanning and extracting three-dimensional meshes of stone artefacts using Micro-CT scanners https://dx.doi.org/10.17504/protocols.io.4r3l24d9qg1y/v2

This is an open access protocol distributed under the terms of the **Creative Commons Attribution License** (https://creativecommons.org/licenses/by/4.0/), which permits unrestricted use, distribution, and reproduction in any medium,

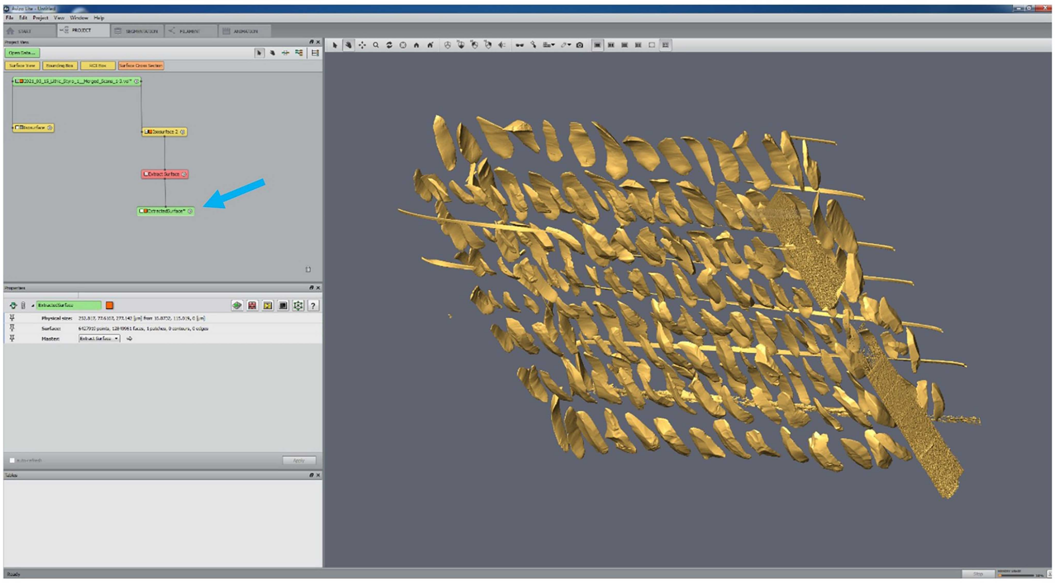

Fig. 21: Extracted surface scan.

25 Save extracted surface model.

25.1 Click on the newly generated‘ExtractedSurface’ label.

25.2 Go to ‘File’ in the menu bar and click ‘Export data as’.

25.3 Navigate to the target folder where you want to save the model.

25.4 Name model.

25.5 Choose file format (e.g., .ply, .stl, .obj).

25.6 Click on ‘Save’ to save the model.

Citation: Dominik GÃÂ¶ldner, Fotios Alexandros Karakostis, Armando Falcucci StyroStone: A protocol for scanning and extracting three-dimensional meshes of stone artefacts using Micro-CT scanners https://dx.doi.org/10.17504/protocols.io.4r3l24d9qg1y/v2

This is an open access protocol distributed under the terms of the **Creative Commons Attribution License** (https://creativecommons.org/licenses/by/4.0/), which permits unrestricted use, distribution, and reproduction in any medium,

26 Save the Avizo project.

26.1 Go to ‘File’ in the menu bar and click ‘Save Project’.

26.2 Select appropriate option in the ‘Save Project Policy’ window (here: ‘Minimize project size’).

26.3 Click ‘Okay’ to confirm selection.

26.4 Navigate to the target folder to save the Avizo project.

26.5 Name project.

26.6 Do not change pre-selected file type (.hx).

26.7 Click ‘Save’ to save the Avizo project.

26.8 A new window might open in Avzio asking to save labels from the project that are necessary to reconstruct the saved Avizo project.

26.9 The target folder should open automatically, if not navigate to it.

26.10 Leave label names as is.

Citation: Dominik GÃÂ¶ldner, Fotios Alexandros Karakostis, Armando Falcucci StyroStone: A protocol for scanning and extracting three-dimensional meshes of stone artefacts using Micro-CT scanners https://dx.doi.org/10.17504/protocols.io.4r3l24d9qg1y/v2

This is an open access protocol distributed under the terms of the **Creative Commons Attribution License** (https://creativecommons.org/licenses/by/4.0/), which permits unrestricted use, distribution, and reproduction in any medium,

26.11 Click ‘Save’ to save the labels.

26.12 Close Avizo Lite.

Part 4 – Cropping extracted surface model to separate Face A and B in Artec Studio

27 Load 3D model into Artec (Fig. 22).

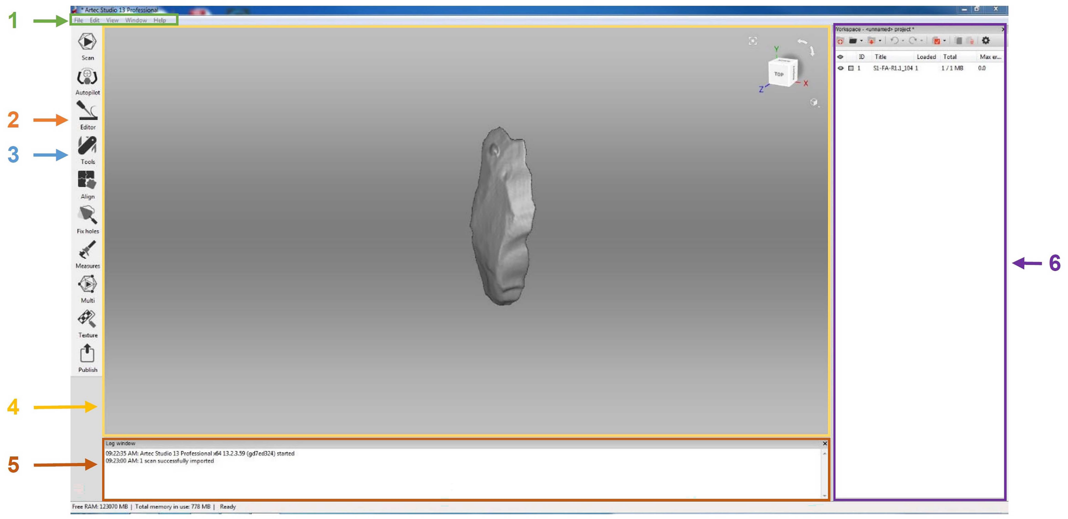

Fig. 22: Artec Studio program overview

27.1 Copy the extracted model file 2 times to get one file for Face A, one for Face B, and one backup file just in case. Name them accordingly.

27.2 Open Artec Studio.

27.3 Click on ‘File’ in the top menu bar and then on Import\scans, meshes or point clouds. Alternatively drag and drop the 3D model file into Artec Studios’ 3D View panel directly from its storage location.

27.4 Navigate to target folder where the extracted 3D model is stored.

27.5 Select the first 3D model file for the creation of Face A.

Citation: Dominik GÃÂ¶ldner, Fotios Alexandros Karakostis, Armando Falcucci StyroStone: A protocol for scanning and extracting three-dimensional meshes of stone artefacts using Micro-CT scanners https://dx.doi.org/10.17504/protocols.io.4r3l24d9qg1y/v2

This is an open access protocol distributed under the terms of the **Creative Commons Attribution License** (https://creativecommons.org/licenses/by/4.0/), which permits unrestricted use, distribution, and reproduction in any medium,

27.6 Click 'Open' to load the 3D model into Artec Studio.

27.7 Press 'NUM 5' to switch to orthographic view.

27.8 Inspect loaded model in Artec Studio (Fig. 23).

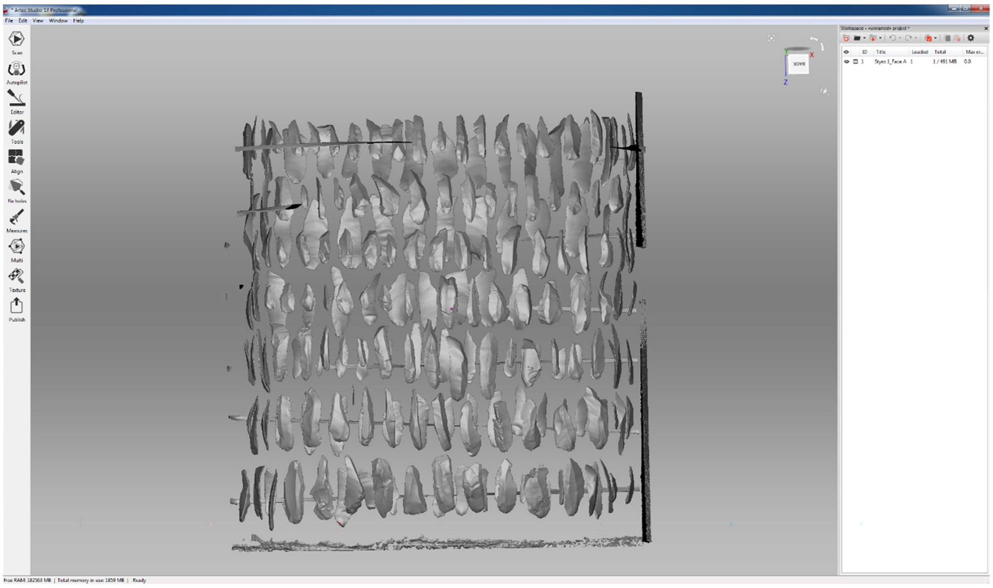

Fig. 23: Loaded 3D model of Styrofoam chunk 1 with both Faces A and B.

28 Deletion of Face B.

28.1 Rotate the model with the left mouse curser in the 3D View Panel so that both faces are parallel to each other like shown in Fig. 24.

Citation: Dominik GÃÂ¶ldner, Fotios Alexandros Karakostis, Armando Falcucci StyroStone: A protocol for scanning and extracting three-dimensional meshes of stone artefacts using Micro-CT scanners https://dx.doi.org/10.17504/protocols.io.4r3l24d9qg1y/v2

This is an open access protocol distributed under the terms of the **Creative Commons Attribution License** (https://creativecommons.org/licenses/by/4.0/), which permits unrestricted use, distribution, and reproduction in any medium,

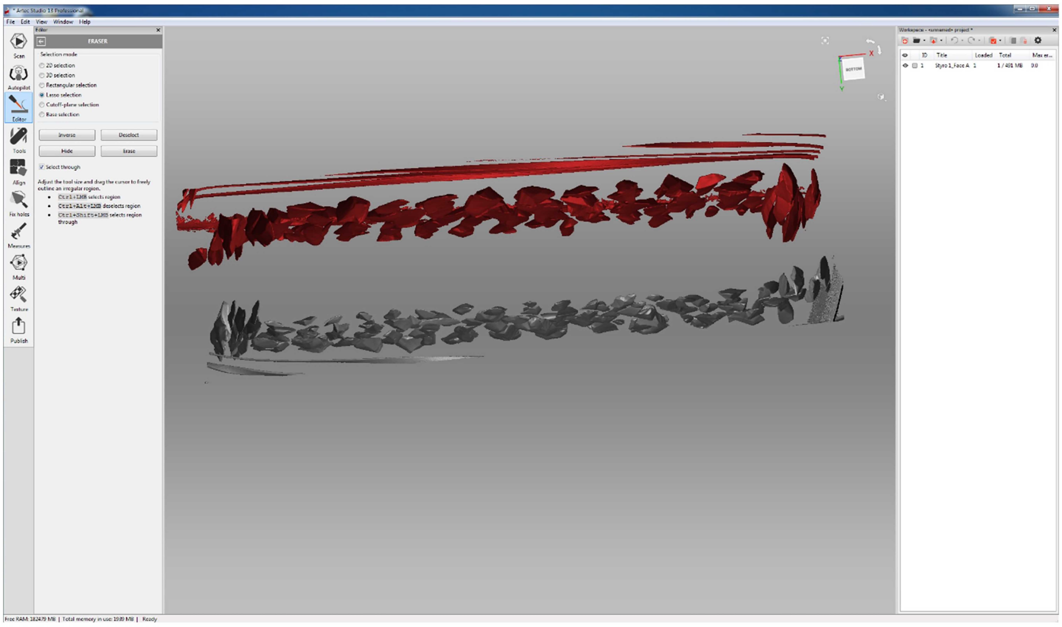

Fig. 24: Selected Face B (red).

28.2 Click on ‘Editor’ in the left Tool Menu Bar.

28.3 Click on ‘Eraser’.

28.4 Select ‘Lasso selection’ in the opened Eraser Panel.

28.5 Press and hold Ctrl while pressing and moving the left cursor over the material from Face B. Selected areas will be highlighted in red (Fig. 24).

28.6 Click ‘Erase’ in the Eraser Panel or press the ‘Delete’ key to delete the selected area (Fig. 25).

Citation: Dominik GÃÂ¶ldner, Fotios Alexandros Karakostis, Armando Falcucci StyroStone: A protocol for scanning and extracting three-dimensional meshes of stone artefacts using Micro-CT scanners https://dx.doi.org/10.17504/protocols.io.4r3l24d9qg1y/v2

This is an open access protocol distributed under the terms of the **Creative Commons Attribution License** (https://creativecommons.org/licenses/by/4.0/), which permits unrestricted use, distribution, and reproduction in any medium,

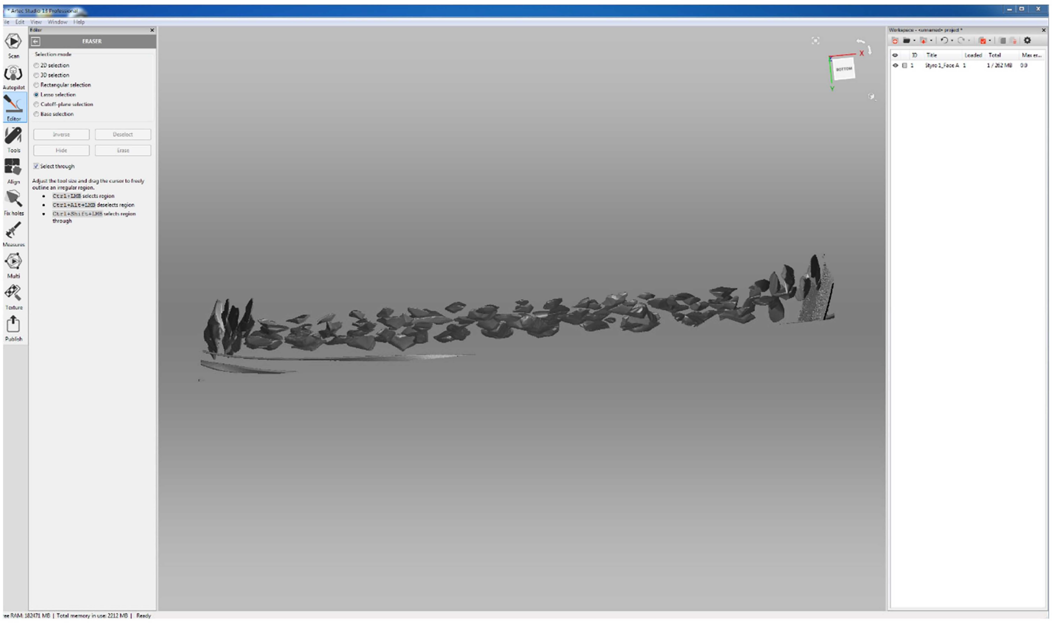

Fig. 25: Remaining Face A after the deletion of Face B.

29 Delete outlying materials.

29.1 If closed, reopen the 'Eraser' menu, and reselect the 'Lasso' tool (see **Step 28.2** to **28.4**).

29.2 To remove the outlying materials, press and hold Ctrl while pressing and dragging the left cursor over the materials. Selected areas will be marked in red (Fig. 26).

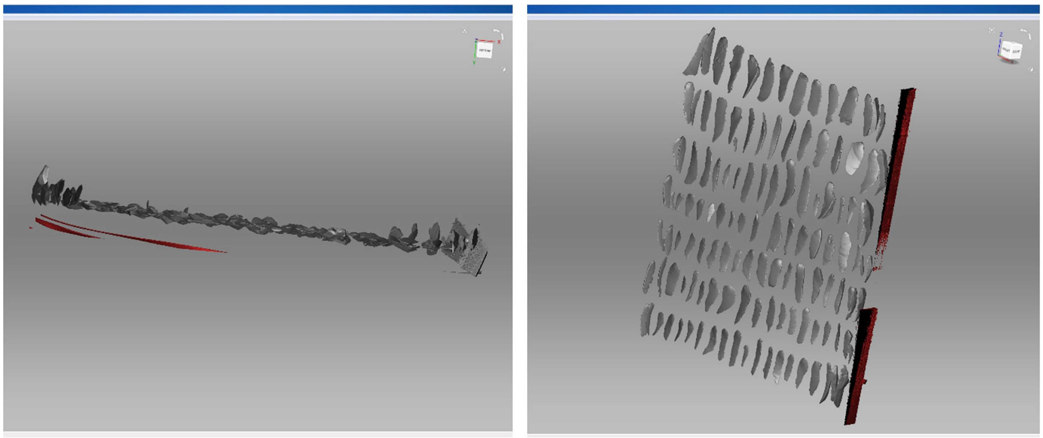

Fig. 26: Selected outlying materials (red).

Citation: Dominik GÃÂ¶ldner, Fotios Alexandros Karakostis, Armando Falcucci StyroStone: A protocol for scanning and extracting three-dimensional meshes of stone artefacts using Micro-CT scanners https://dx.doi.org/10.17504/protocols.io.4r3l24d9qg1y/v2

This is an open access protocol distributed under the terms of the **Creative Commons Attribution License** (https://creativecommons.org/licenses/by/4.0/), which permits unrestricted use, distribution, and reproduction in any medium,

29.3 If it is not possible to select all outlying materials at once, repeat **Step 29.2** until only the stone artefacts remain. It might help to rotate the entire scan to get a better viewpoint.

30 Save the isolated and cleaned Face (Fig. 27).

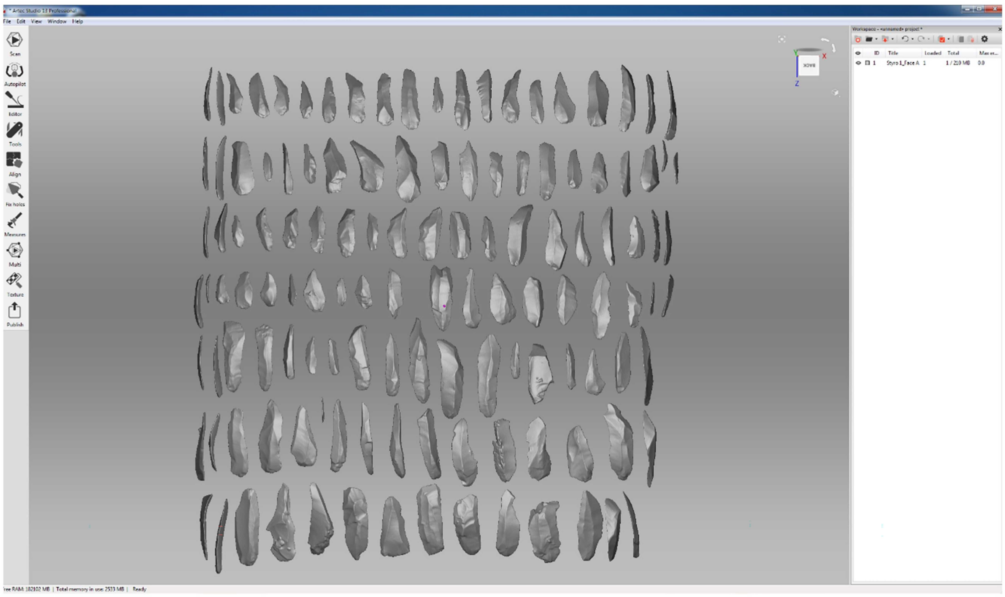

Fig. 27: Isolated and cleaned Face A.

30.1 Go to 'File' in the menu bar and click on 'Export\Meshes'.

30.2 Navigate to the target folder in which to save the model.

30.3 Name model (e.g., "Styro 1_Face A_Cropped and Cleaned").

30.4 Choose file format (e.g., .ply, .stl, .obj, or .wrl).

30.5 Click on 'Save' to save the model.

Citation: Dominik GÃÂ¶ldner, Fotios Alexandros Karakostis, Armando Falcucci StyroStone: A protocol for scanning and extracting three-dimensional meshes of stone artefacts using Micro-CT scanners https://dx.doi.org/10.17504/protocols.io.4r3l24d9qg1y/v2

This is an open access protocol distributed under the terms of the **Creative Commons Attribution License** (https://creativecommons.org/licenses/by/4.0/), which permits unrestricted use, distribution, and reproduction in any medium,

30.6 In the storage folder duplicate Face A file by copying it to create a backup file. Name it accordingly (e.g., “Styro 1_Face A_Cropped and Cleaned_Backup”).

31 Repeat the workflow for Face B with the copied version of the extracted Avizo file from **Step 27.1.**

Part 5 – Cropping Face A to separate the lines in Artec Studio

32 Create a folder where the individual lines are stored and name it accordingly.

33 Open the cropped and cleaned model of Face A in Artec Studio.

34 Crop the lines to separate them.

34.1 Rotate the model with the left mouse curser in the 3D View Panel as shown in Fig. 27. Remember to position the scan so that their order corresponds to their actual order, i.e., the first artefact in the model must be the first on in the list, so it is placed on the upper left. Also remember to look at the scan from the correct side. The scan can also be oriented using the reference pictures.

34.2 Click on ‘Editor’ in the left Tool Menu Bar.

34.3 Click on ‘Eraser’.

34.4 Select ‘Lasso selection’ in the Eraser Panel.

34.5 Press and hold Ctrl while outlining all rows except the one that you want to separate. Selected areas will be marked in red (Fig. 28).

Citation: Dominik GÃÂ¶ldner, Fotios Alexandros Karakostis, Armando Falcucci StyroStone: A protocol for scanning and extracting three-dimensional meshes of stone artefacts using Micro-CT scanners https://dx.doi.org/10.17504/protocols.io.4r3l24d9qg1y/v2

This is an open access protocol distributed under the terms of the **Creative Commons Attribution License** (https://creativecommons.org/licenses/by/4.0/), which permits unrestricted use, distribution, and reproduction in any medium,

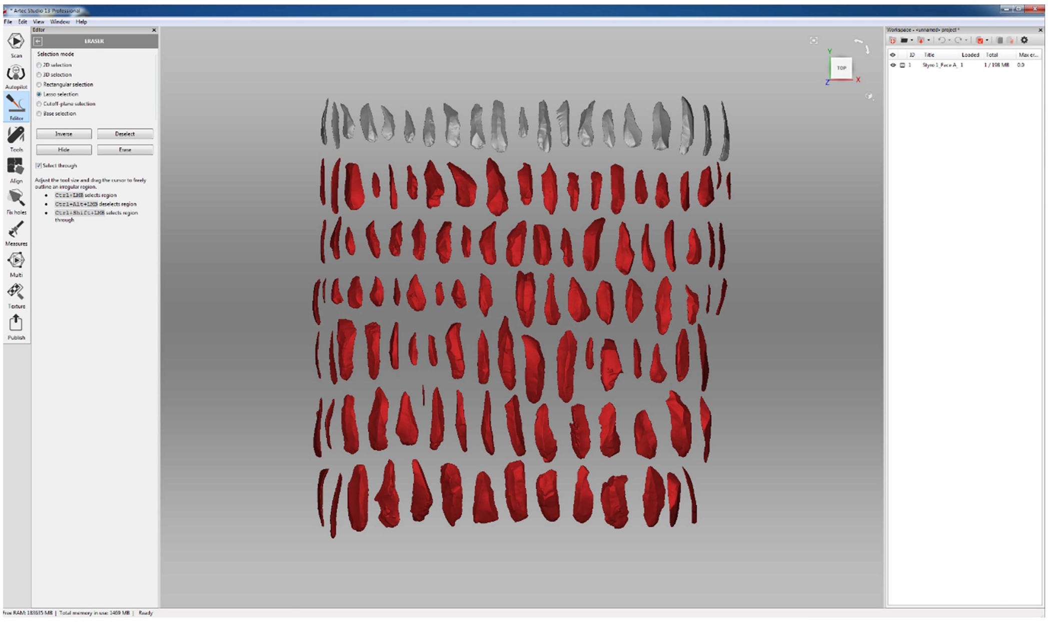

Fig. 28: Selected lines that will be deleted to separate the first line.

34.6 Click ‘Erase’ in the Eraser Panel or press the ‘Delete’ key to delete the selected rows.

34.7 Alternatively, you can select the line that you want to separate and then inverse the selection with the ‘Inverse’ button in the Eraser Panel. To delete the selection, apply **Step 34.6**.

35 Save the separated line (Fig. 29).

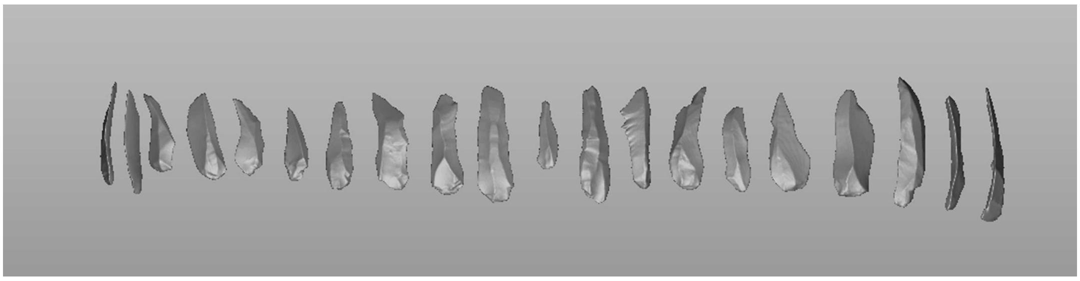

Fig. 29: Cropped line 1.

35.1 Click on ‘File’ in the top menu bar.

Citation: Dominik GÃÂ¶ldner, Fotios Alexandros Karakostis, Armando Falcucci StyroStone: A protocol for scanning and extracting three-dimensional meshes of stone artefacts using Micro-CT scanners https://dx.doi.org/10.17504/protocols.io.4r3l24d9qg1y/v2

This is an open access protocol distributed under the terms of the **Creative Commons Attribution License** (https://creativecommons.org/licenses/by/4.0/), which permits unrestricted use, distribution, and reproduction in any medium,

35.2 Click on ‘Export as’.

35.3 Navigate to the target folder.

35.4 Name the cropped line accordingly (e.g., “Styro 1_Line 1”).

35.5 Select the file format (e.g., .ply, .stl. etc.).

35.6 Press ‘Okay’ to export the cropped line.

Important: Do not save and close the entire project. All deleted lines will be lost otherwise! See next step.

36 Recall the deleted lines to continue separating the next line.

36.1 Press Ctrl and Z to undo the deletion or click on ‘Edit’ in the top menu bar and press on ‘Undo’.

37 Separate the next line.

37.1 Apply **Steps 34.2 to 34.6** for the next line.

37.2 Apply **Steps 35.1 to 35.6** to save the newly separated line. Remember to name it accordingly (e.g., “Styro 1_Line 2” for the second line and so on).

38 Repeat all steps for each line individually.

Citation: Dominik GÃÂ¶ldner, Fotios Alexandros Karakostis, Armando Falcucci StyroStone: A protocol for scanning and extracting three-dimensional meshes of stone artefacts using Micro-CT scanners https://dx.doi.org/10.17504/protocols.io.4r3l24d9qg1y/v2

This is an open access protocol distributed under the terms of the **Creative Commons Attribution License** (https://creativecommons.org/licenses/by/4.0/), which permits unrestricted use, distribution, and reproduction in any medium,

Part 6 – Cropping each stone artefact from the lines in Artec Studio

39 Create a backup copy of each of the separated lines and name them accordingly.

40 Create folders for each line and name it accordingly.

41 Move the separated lines into their respective folder.

42 Open the first line with Artec Studio.

43 Crop the stone artefacts to separate them.

43.1 Rotate the line model with the left mouse curser in the 3D View Panel as shown in Fig. 30. Remember to position the scan so that the artefacts in the model correspond to their actual order, i.e., the first artefact in the model must be the first on in the list, so it is positioned in the upper left. Also remember to look at the scan from the correct side. This can be done by orienting the scan using the reference pictures.

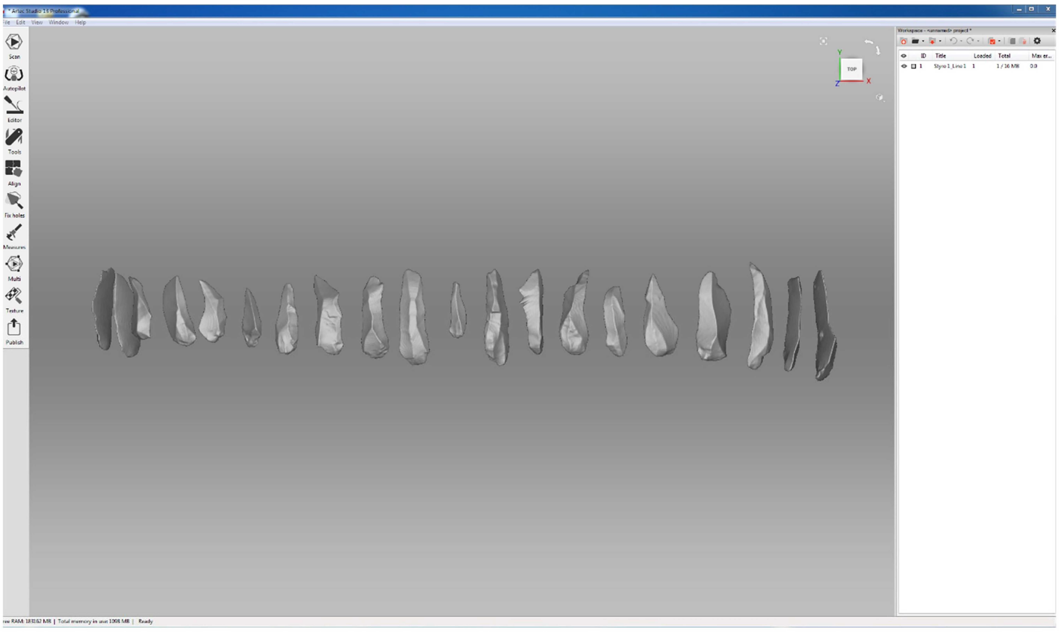

Fig. 30: Loaded line 1 in correct position.

Citation: Dominik GÃÂ¶ldner, Fotios Alexandros Karakostis, Armando Falcucci StyroStone: A protocol for scanning and extracting three-dimensional meshes of stone artefacts using Micro-CT scanners https://dx.doi.org/10.17504/protocols.io.4r3l24d9qg1y/v2

This is an open access protocol distributed under the terms of the **Creative Commons Attribution License** (https://creativecommons.org/licenses/by/4.0/), which permits unrestricted use, distribution, and reproduction in any medium,

43.2 Click on ‘Editor’ in the left Tool Menu Bar.

43.3 Click on ‘Eraser’.

43.4 Select ‘Lasso selection’ in the Eraser Panel.

43.5 Press and hold Ctrl while selecting all artefacts but the one that you want to separate. Selected artefacts will be highlighted red (Fig. 31).

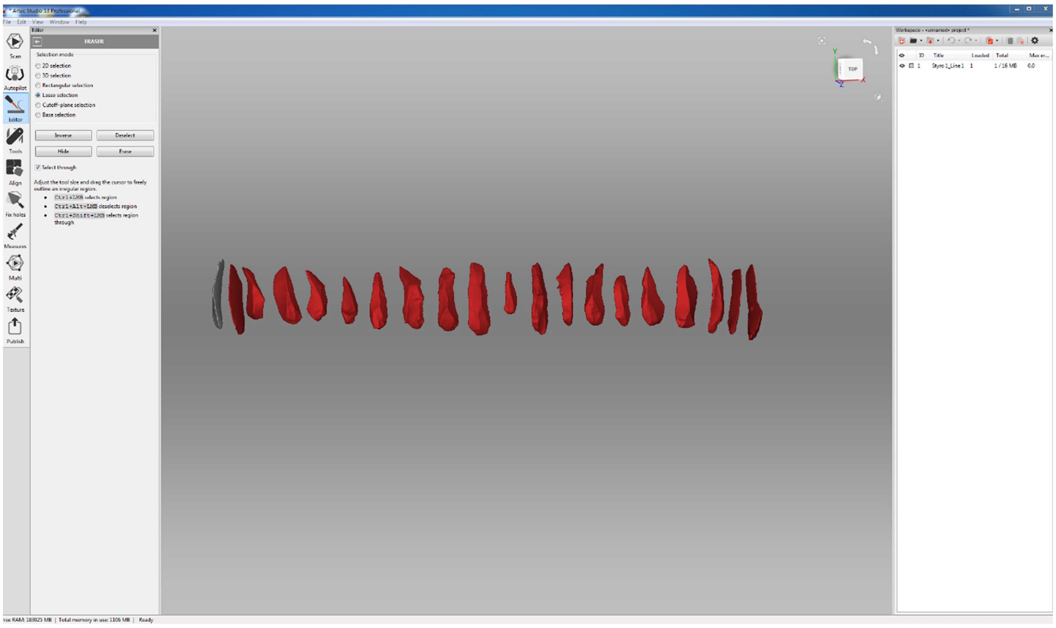

Fig. 31: Selected artefacts which will be deleted to separate the first one.

43.6 Click ‘Erase’ in the Eraser Panel or press the ‘Delete’ key to delete the selected artefacts.

43.7 Alternatively, you can also select only the artefact that you want to separate and then inverse the selection with the ‘Inverse’ button in the Eraser Panel. To delete the selection, apply **Step 43.6**.

Export and save the separated artefact (Fig. 32).

Citation: Dominik GÃÂ¶ldner, Fotios Alexandros Karakostis, Armando Falcucci StyroStone: A protocol for scanning and extracting three-dimensional meshes of stone artefacts using Micro-CT scanners https://dx.doi.org/10.17504/protocols.io.4r3l24d9qg1y/v2

This is an open access protocol distributed under the terms of the **Creative Commons Attribution License** (https://creativecommons.org/licenses/by/4.0/), which permits unrestricted use, distribution, and reproduction in any medium,

44

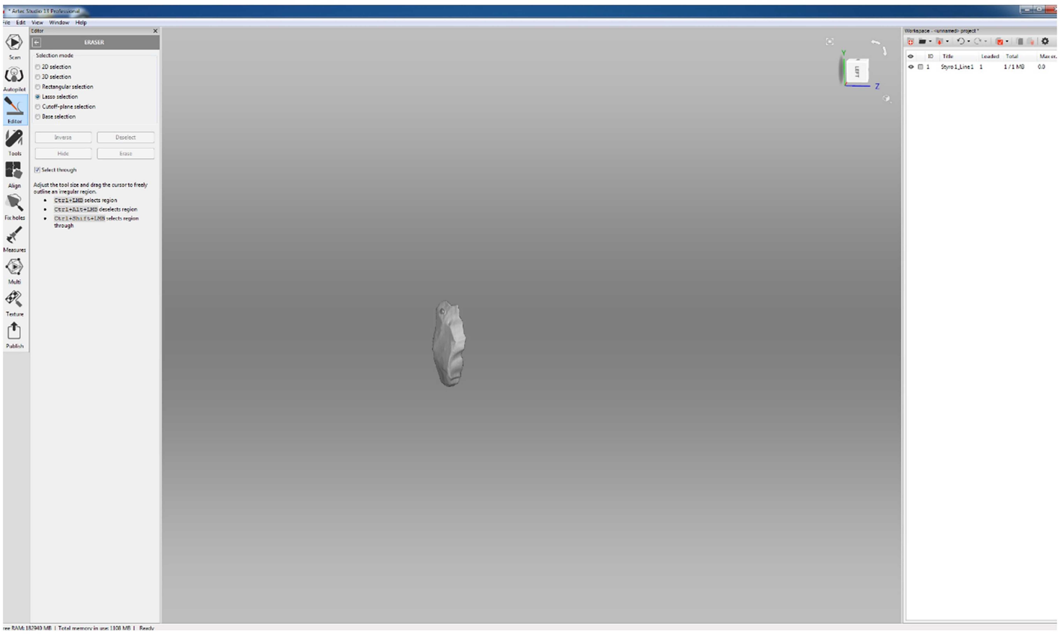

Fig. 32: Cropped and isolated artefact. Note that the model was rotated before the screenshot was captured. It is the same non-selected artefact as in Fig. 31.

44.1 Click on ‘File’ in the top menu bar.

44.2 Click on ‘Export as’.

44.3 Navigate to the target folder.

44.4 Name the cropped artefact with the actual artefact ID.

44.5 It might also be useful to add information on the exact location within the Styrofoam scan to trace potential errors. A possible location ID which can be added either before or after the actual artefact ID in the file name could look like this:

S1-FA-L1.1_10401

Citation: Dominik GÃÂ¶ldner, Fotios Alexandros Karakostis, Armando Falcucci StyroStone: A protocol for scanning and extracting three-dimensional meshes of stone artefacts using Micro-CT scanners https://dx.doi.org/10.17504/protocols.io.4r3l24d9qg1y/v2

This is an open access protocol distributed under the terms of the **Creative Commons Attribution License** (https://creativecommons.org/licenses/by/4.0/), which permits unrestricted use, distribution, and reproduction in any medium,

S1 stands for the first Styrofoam scan, **FA** stands for Face A, **L1.1** stands for the first line – first artefact. The last number **10401** represents the artefact ID. It might also be useful to include this information in the artefact database.

44.6 Select the file format (e.g., .ply, .stl. etc.). Remember to also export the model as a .wrl file, as AGMT-3D only can import this file format. If the R package geomorph will be used subsequentially, the models must be saved as non-binary ply files (see below).

44.7 Press 'Okay' to export the cropped artefact.

Note: Do not save and close the entire project. All deleted lines will be lost otherwise! See next step.

45 Recall the deleted artefacts to continue separating the next one.

45.1 Press Ctrl + Z to undo the deletion or click on 'Edit' in the top menu bar.

46 Separate the next artefact.

46.1 Apply **Steps 43.2 to 43.6** for the next artefact.

46.2 Apply **Steps 44.1 to 44.7** to export and save the newly separated artefacts. Remember to name it accordingly (see **Step 44.5**).

47 Repeat all steps for each artefact individually.

48 Repeat these steps for all lines.

Part 7 – Virtually control measurements in MeshLab

49 **Note:** It might be useful to take control measurements for time to time, e.g., of the first and last artefact in a line and a few random artefacts elsewhere. This helps to check whether the line is exactly positioned and whether any artefacts were accidently skipped.

Citation: Dominik GÃÂ¶ldner, Fotios Alexandros Karakostis, Armando Falcucci StyroStone: A protocol for scanning and extracting three-dimensional meshes of stone artefacts using Micro-CT scanners https://dx.doi.org/10.17504/protocols.io.4r3l24d9qg1y/v2

This is an open access protocol distributed under the terms of the **Creative Commons Attribution License** (https://creativecommons.org/licenses/by/4.0/), which permits unrestricted use, distribution, and reproduction in any medium,

If all artefacts are measured, errors in the database could also be cross-checked. However, with larger sample sizes, this may take a lot of time. Linear measurements (e.g., maximum length of the artefact) can easily be taken in MeshLab (Cignoni et al. 2008) then compared with physically measured variables in the main database. For any subsequent intra- or interobserver error and methodological evaluations, it might be useful to store the virtual control measurements in an extra column of the main database. To save time, this task can easily be combined with the export of the models to non-binary ply. files if necessary for subsequent analysis in the R package *geomorph* (see below).

Open MeshLab.

50 To load the first artefact into MeshLab, drag the model file and drop it into the 3D View Panel of MeshLab. Alternatively, click on ‘File’ in the top menu bar and choose ‘Import Mesh’, then navigate to the target folder and open the desired model by pressing ‘Open’ (Fig. 33).

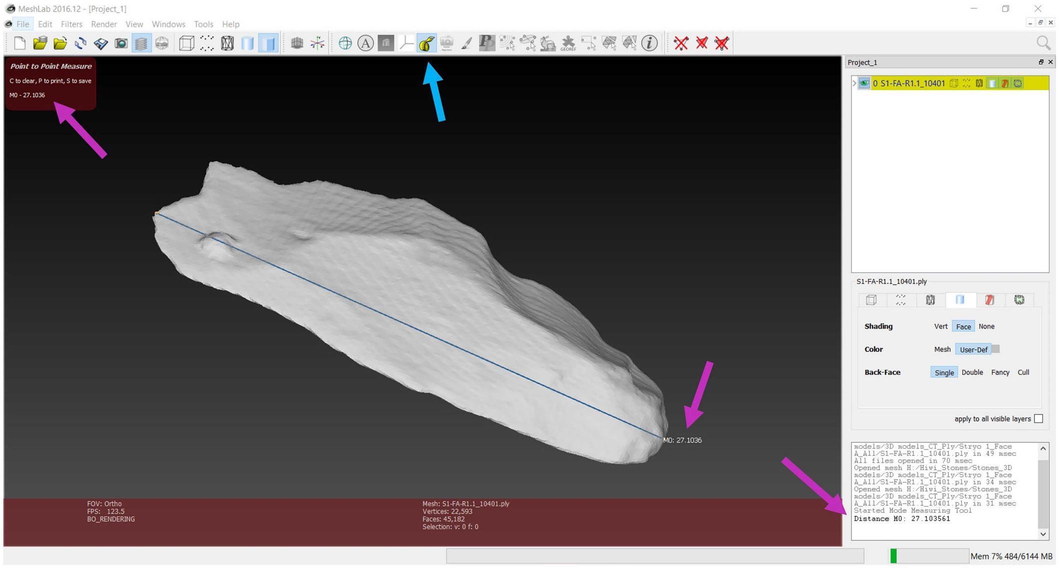

Fig. 33: Artefact opened in MeshLab. The maximum length of the artefact was measured with the measuring tool. The measuring tool is still activated (no tracking ball visible). The measurement value is shown beside the right-most point, in the ‘Point to Point Measure’ panel in the upper left corner of the 3D View Panel, and in the lower right Log Panel (pink arrows).

51 Press ‘5’ on the keyboard to switch to orthographic view.

52 Click on the ‘Point to Point Measure Tool’ in the Tool bar above the 3D View Panel (blue arrow in Fig. 33). In this mode, the tracking ball will vanish, making it impossible to rotate the model (but see note within **Step 54**).

53 Click with the left mouse curser on the starting point of the measurement.

Citation: Dominik GÃÂ¶ldner, Fotios Alexandros Karakostis, Armando Falcucci StyroStone: A protocol for scanning and extracting three-dimensional meshes of stone artefacts using Micro-CT scanners https://dx.doi.org/10.17504/protocols.io.4r3l24d9qg1y/v2

This is an open access protocol distributed under the terms of the **Creative Commons Attribution License** (https://creativecommons.org/licenses/by/4.0/), which permits unrestricted use, distribution, and reproduction in any medium,

54 Click with the left mouse curser on the end point of the measurement to finish the measurement.

Note: If it is necessary to rotate the model, place the first point (**Step 53**) and then press 'Esc' on the keyboard to temporally leave the measuring tool. When the tool is disabled, the tracking ball will reappear. Rotate the model accordingly and then press 'Esc' to return to the measuring mode.

55 After the measurement is taken, the model can be rotated to check the placements and evaluate the measurement. Redo if necessary, by double clicking on the 'Measuring Tool' in the Tool bar menu and repeating **Steps 52 to 54**.

56 The measurement will appear beside the end point of the measurement, as well as in the 'Point to Point Measure' panel in the upper left corner of the 3D View Panel and in the lower right Log Panel (pink arrows in Fig. 33).

57 Copy the measurement value to the main database and/or compare with previous measurements to control for error.

Part 8 – Artec scanning of larger artefacts

58 **General tips for Artec scanning:**

- The lighting in the room should be uniform, cold and diffuse;
- Try to reduce areas of shading during the scanning process;
- Use a scanning speed of 8 fps (frames per second) and a depth of 170. Both can be adjusted in the Scan menu in Artec Studio;
- For dark objects set a higher brightness.

Note: Calculation of surface texture colour is not necessary for most analyses. In order to save time and computation capacity, this step is not included here.

Set up the scanner and workspace.

59 Boot the scanning computer.

60 Set up the Calibration equipment according to the Artec manual (http://docs.artec-group.com/as/13/en).

61 Open the Artec Studio software.

62 Calibrate the Artec scanner according to the official instructions of the Artec manual (http://docs.artec-group.com/as/13/en).

Citation: Dominik GÃÂ¶ldner, Fotios Alexandros Karakostis, Armando Falcucci StyroStone: A protocol for scanning and extracting three-dimensional meshes of stone artefacts using Micro-CT scanners https://dx.doi.org/10.17504/protocols.io.4r3l24d9qg1y/v2

This is an open access protocol distributed under the terms of the **Creative Commons Attribution License** (https://creativecommons.org/licenses/by/4.0/), which permits unrestricted use, distribution, and reproduction in any medium,

63 Put the calibration equipment away.

64 Set up the turntable. We recommend using a white turntable or cover a turntable with white cardboard. As suggested by Archer et al. (2021), red and blue circles should be randomly marked on the turntable's surface to enhance tracking during scanning (Fig. 34).

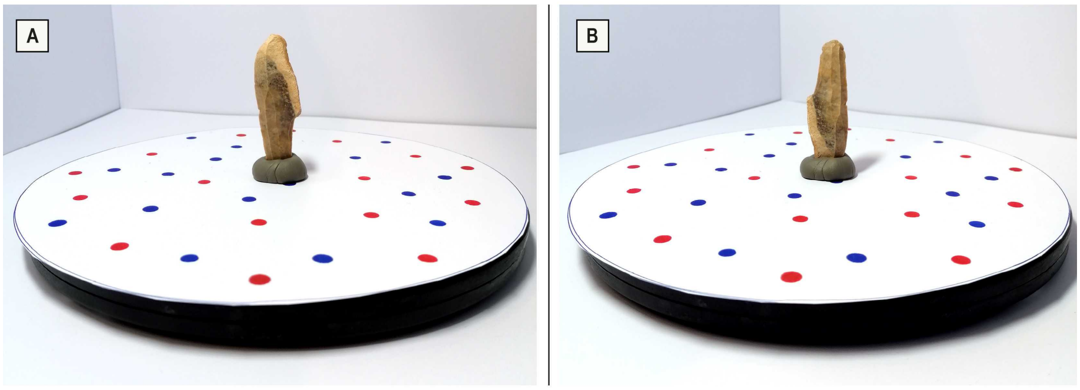

Fig. 35: Lithic artefact fixed on a manual turntable using modeling clay. The turntable was covered with a white cardboard with randomly marked blue and red dots for better tracking. Left (A): Blade tool with proximal side down for the first scanning round. Right image (B): The same blade rotated of 180 degrees (distal side down) after the completion of the first scan.

65 Place the artefact on the turntable.

Use modeling clay to secure the lithic vertically on the table so that it will not move during the scanning process.

66 Start the pre-scanning process by pressing the button on the back of the Artec handle upwards.

67 Find the optimal distance between the scanner and the artefact, indicated by the range on the left side of the scanning computer screen and the color of the objects in view. Green means optimal distance, blue too far away, and red to close.

68 Press the button on the back of the Artec handle upwards again to start the scanning process.

69 Hold the scanner in one hand while slowly but steadily spinning the turntable with the other hand. Keep your eyes on the scanning computer monitor to watch the scanning progress, navigate the scanning directions and to keep a constant scanning speed.

Citation: Dominik GÃÂ¶ldner, Fotios Alexandros Karakostis, Armando Falcucci StyroStone: A protocol for scanning and extracting three-dimensional meshes of stone artefacts using Micro-CT scanners https://dx.doi.org/10.17504/protocols.io.4r3l24d9qg1y/v2

This is an open access protocol distributed under the terms of the **Creative Commons Attribution License** (https://creativecommons.org/licenses/by/4.0/), which permits unrestricted use, distribution, and reproduction in any medium,

70 When the scan is completed stop the scanning process by pressing the button on the back of the Artec handle downwards.

70.1 Artec Studio will automatically run the ‘Fine registration’.

71 Turn the artefact upside down on the table using the modeling clay (**Step 65**) to capture the side that could not be scanned in the first round. Sometimes objects need to be scanned from more than two sides to capture all areas.

72 Repeat **Step 66 to Step 70.1** to scan the second side.

73 Removing the base and outlying material.

73.1 Open the ‘Editor’ menu in Artec Studio.

73.2 Open the ‘Eraser’ tool.

73.3 Select the ‘Lasso selection’ tool.

73.4 While pressing the left mouse button, move the scanned artefact model into a position where the base can easily be removed without deleting to much of the artefact itself, i.e., by placing the model so that the flat base part is horizontally aligned and only appears as a thin line on the screen display.

73.5 Press and hold Ctrl + left mouse button to apply the lasso tool, then drag it around the base part to select it. Selected areas will be marked in red.

73.6 Press ‘Delete’ on the keyboard or ‘Erase’ in ‘Eraser’ menu to delete the selected base.

Citation: Dominik GÃÂ¶ldner, Fotios Alexandros Karakostis, Armando Falcucci StyroStone: A protocol for scanning and extracting three-dimensional meshes of stone artefacts using Micro-CT scanners https://dx.doi.org/10.17504/protocols.io.4r3l24d9qg1y/v2

This is an open access protocol distributed under the terms of the **Creative Commons Attribution License** (https://creativecommons.org/licenses/by/4.0/), which permits unrestricted use, distribution, and reproduction in any medium,

73.7 Repeat **Step 73.4 to Step 73.6** for the second scan. Disable the first scan and enable the second scan in the model scan menu on the right side. If you have more than two scans, repeat the base removal steps for all of them.

73.8 Close the 'Eraser tool menu. This will also close the 'Editor' menu.

74 Align the sub-scans.

74.1 Open the 'Align' menu.

74.2 Press 'Auto-alignment'.

In most cases automatic alignment should work successfully. However, when the program is not able to detect multiple matching points itself, this must be done manually. If the program misaligned two or more sub-scans, cancel the result.

To manually align sub-scans, use the options to place matching pairs between each scan. If there are more than two sub-scans, align one after the other. With this function three to five pairs of homologous points must be placed on both sub-scans. Choose points that are characteristic and can easily be identified on both sub-scans. The placement does not have to be extremely perfect. The software will detect the right point itself.

74.3 Press 'Apply' to complete the process and to leave the 'Align' menu.

75 Finish the model.

75.1 Open the 'Tools' menu.

75.2 Apply 'Global registration'.
(Use default settings: Geometry and texture; Key frame ratio: 0.1; Focus on geometry disabled; Search features within, mm: 50).

75.3 Apply 'Sharp fusion'.

Citation: Dominik GÃÂ¶ldner, Fotios Alexandros Karakostis, Armando Falcucci StyroStone: A protocol for scanning and extracting three-dimensional meshes of stone artefacts using Micro-CT scanners https://dx.doi.org/10.17504/protocols.io.4r3l24d9qg1y/v2

This is an open access protocol distributed under the terms of the **Creative Commons Attribution License** (https://creativecommons.org/licenses/by/4.0/), which permits unrestricted use, distribution, and reproduction in any medium,

(Use default settings: 3D resolution, mm: 1; Fill holes: With radius smaller ...; Hole radius (max), mm: 5; Remove targets disabled).

75.4 Apply 'Hole filling'.
(use default settings: Hole perimeter (max), mm: 100)

75.5 Apply 'Small-object filter'.
(Use default settings: Remove surfaces: All except largest)

76 Save the project.

76.1 Click on 'File' in the top menu bar.

76.2 Click on 'Save project as ...'.

76.3 Navigate to and select the target folder.

76.4 Name the project with the actual ID of the artefact.

76.5 Press 'Okay' to finish this step.

77 Export model as ply file.

77.1 Click on 'File' in the top menu bar.

77.2 Click on 'Export'.

Citation: Dominik GÃÂ¶ldner, Fotios Alexandros Karakostis, Armando Falcucci StyroStone: A protocol for scanning and extracting three-dimensional meshes of stone artefacts using Micro-CT scanners https://dx.doi.org/10.17504/protocols.io.4r3l24d9qg1y/v2

This is an open access protocol distributed under the terms of the **Creative Commons Attribution License** (https://creativecommons.org/licenses/by/4.0/), which permits unrestricted use, distribution, and reproduction in any medium,

77.3 Click on ‘Meshes’.

77.4 Select .ply as file format.

77.5 Save the exported ply mesh following the instructions in **Step 76.3 to Step 76.5.**

Part 9 – Export meshes as non-binary ply models for successive analysis in *geomorph*

78 **Note:** As the R (R Core Team 2020) package geomorph (Adams et al. 2021) requires non-binary ply models, the program MeshLab (Cignoni et al. 2008) can be used to export and save the separated artefacts in this required format.

Open MeshLab.

79 To load the first artefact into MeshLab, drag the model file and drop it into the 3D View Panel of MeshLab. Alternatively, click on ‘File’ in the top menu bar and choose ‘Import Mesh’, then navigate to the target folder and choose and open the respective model by pressing ‘Open’ (Fig. 35)

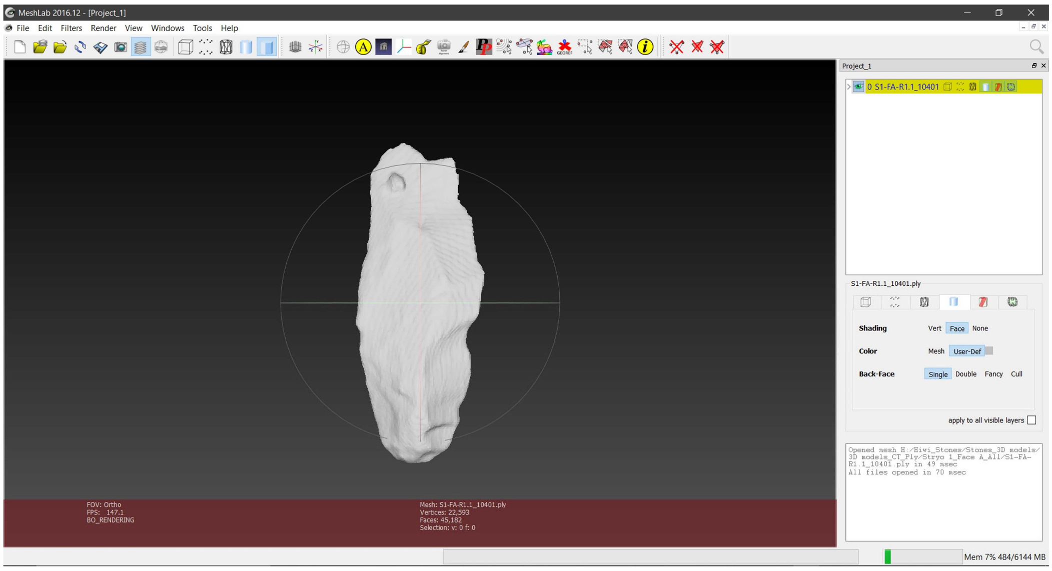

Fig. 35: Artefact opened in MeshLab.

80 Press ‘5’ on the keyboard to switch to orthographic view.

Citation: Dominik GÃÂ¶ldner, Fotios Alexandros Karakostis, Armando Falcucci StyroStone: A protocol for scanning and extracting three-dimensional meshes of stone artefacts using Micro-CT scanners https://dx.doi.org/10.17504/protocols.io.4r3l24d9qg1y/v2

This is an open access protocol distributed under the terms of the **Creative Commons Attribution License** (https://creativecommons.org/licenses/by/4.0/), which permits unrestricted use, distribution, and reproduction in any medium,

81 Press Ctrl + Shift + E to export the model or click on File\Export Mesh As...

82 Navigate to the target folder.

83 Select file format (note: if you change the current file format, it might be necessary to (partially) rename the file).In the ‘Choose Saving Options for *ID*’ panel, disable ‘Binary encoding’ (Fig. 36).

84 Press ‘Open’.

85 In the ‘Choose Saving Options for *ID*’ panel, disable ‘Binary encoding’ (Fig. 36).

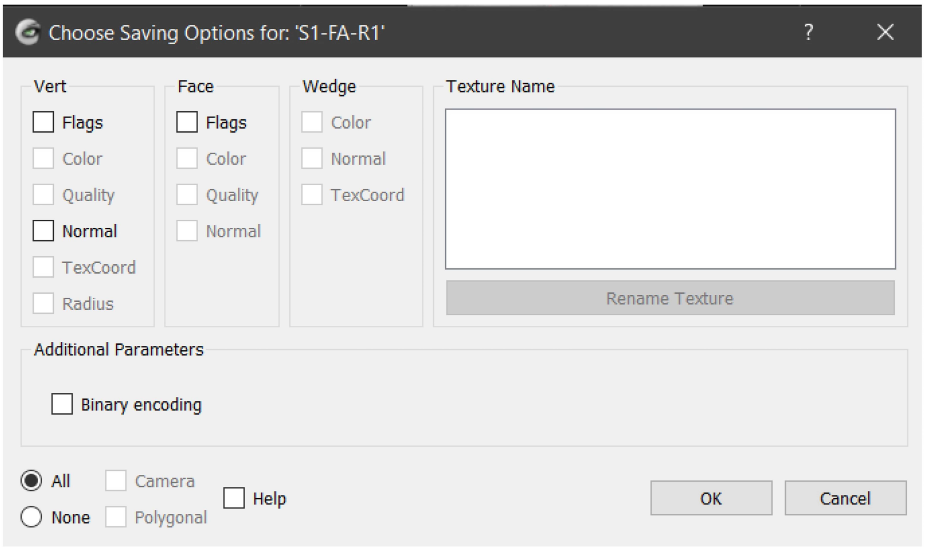

Fig. 37: ‘Choose Saving Options for ‘ID’’ panel with disabled ‘Binary encoding’.

86 Press ‘Okay’.

Citation: Dominik GÃÂ¶ldner, Fotios Alexandros Karakostis, Armando Falcucci StyroStone: A protocol for scanning and extracting three-dimensional meshes of stone artefacts using Micro-CT scanners https://dx.doi.org/10.17504/protocols.io.4r3l24d9qg1y/v2

This is an open access protocol distributed under the terms of the **Creative Commons Attribution License** (https://creativecommons.org/licenses/by/4.0/), which permits unrestricted use, distribution, and reproduction in any medium,
